# Evolution in chronic cold: varied loss of cellular response to heat in Antarctic notothenioid fish

**DOI:** 10.1186/s12862-018-1254-6

**Published:** 2018-09-19

**Authors:** Kevin T. Bilyk, Luis Vargas-Chacoff, C.-H.Christina Cheng

**Affiliations:** 1 Department of Biology, Western Kentucky University, 1906 College Heights Blvd, Bowling Green, KY 42101 USA; 2 School of Integrative Biology, University of Illinois, Urbana-Champaign, USA; 3 Instituto de Ciencias Marinas y Limnológicas, Laboratorio de Fisiología de Peces, Centro Fondap de Investigación de Altas Latitudes (IDEAL), Universidad Austral de Chile, Valdivia, Chile

**Keywords:** Antarctic fish, Notothenioid, Cold-specialization, Cellular stress response, Stenothermal, Thermal stress, RNA Seq, Gene expression

## Abstract

**Background:**

Confined within the freezing Southern Ocean, the Antarctic notothenioids have evolved to become both cold adapted and cold specialized. A marked signature of cold specialization is an apparent loss of the cellular heat shock response (HSR). As the HSR has been examined in very few notothenioid species to-date, it remains unknown whether HSR loss pervades the Antarctic radiation, or whether the broader cellular responses to heat stress has sustained similar loss. Understanding the evolutionary status of these responses in this stenothermal taxon is crucial for evaluating its adaptive potential to ocean warming under climate change.

**Results:**

In this study, we used an acute heat stress protocol followed by RNA-Seq analyses to study the evolution of cellular-wide transcriptional responses to heat stress across three select notothenioid lineages - the basal temperate and nearest non-Antarctic sister species *Eleginops maclovinus* serving as ancestral proxy, the cryopelagic *Pagothenia borchgrevinki* and the icefish *Chionodraco rastrospinosus* representing cold-adapted red-blooded and hemoglobinless Antarctic notothenioids respectively. *E. maclovinus* displayed robust cellular stress responses including the ER Unfolded Protein Response and the cytosolic HSR, cementing the HSR as a plesiomorphy that preceded Antarctic notothenioid radiation. While the transcriptional response to heat stress was minimal in *P. borchgrevinki*, *C. rastrospinosus* exhibited robust responses in the broader cellular networks especially in inflammatory responses despite lacking the classic HSR and UPR.

**Conclusion:**

The disparate patterns observed in these two archetypal Antarctic species indicate the evolutionary status in cellular ability to mitigate acute heat stress varies even among Antarctic lineages, which may affect their adaptive potential in coping with a warming world.

**Electronic supplementary material:**

The online version of this article (10.1186/s12862-018-1254-6) contains supplementary material, which is available to authorized users.

## Background

Evolution in the unrelenting cold of the isolated Southern Ocean has profoundly shaped the biology of the endemic Antarctic notothenioid fishes. The strong selective pressures imposed by the freezing, icy waters have driven innovative evolutionary adaptations that enable life at subzero temperatures, most notably the antifreeze proteins that protect the fish from death by inoculative freezing [1, 2]. At the same time, the steady cold would reduce selective pressures on maintaining functional abilities to mitigate challenges from large thermal fluctuations. Under these dual influences, Antarctic notothenioid fishes today are not only highly cold-adapted but also cold-stenothermal, exhibiting greatly reduced heat tolerance compared to non-polar fishes [3–6]. Mirroring the reduced organismal thermal tolerance in these fishes, their cellular response seems similarly compromised as they appear to have lost the near-universal heat shock response (HSR) first noted in their inability to synthesize heat shock proteins in response to acute heat stress [7]. Thus far, cellular heat stress response has been examined for a limited number of Antarctic notothenioid species, thus it is unknown whether HSR had become uniformly lost across this large taxon. Also, apart from HSR, the full nature and extent of broader cellular responses, which are normally activated by heat shock, remain incompletely understood.

At the cellular level, environmental stresses elicit a generally conserved suite of responses that act to mitigate damage and stabilize cellular homeostasis [8, 9]. Denaturing stresses like heat shock set off compartment specific responses within the cell. Most well-known is the cytoplasmic HSR, regulated by members of the heat shock transcription factor (HSF) family. When activated, HSFs induce expression of diverse molecular chaperones amid the larger network of genes whose expression is triggered by general cellular stress [10, 11]. Concurrently, the accumulation of denatured, unfolded, and misfolded proteins within the lumen of the endoplasmic reticulum (ER) leads to ER stress and activates ER-specific molecular chaperones in the Unfolded Protein Response (UPR) [12, 13].

In addition to the molecular chaperones, environmental stresses and cellular stimuli instigate a broad cellular stress response, coordinated through highly conserved cell signaling pathways and gene regulatory networks [14]. Among them, the stress activated MAPK (mitogen-activated protein kinase) cascades are particularly well described [15, 16]. These act to instigate widespread changes in gene expression towards either restoring cellular homeostasis or triggering apoptosis when cellular damage exceeds the capacity for recovery. Many of these activities occur through the rapid and transient induction of a functionally diverse set of genes, including many potent transcription factors that act as gateways to a broader genome-wide response [17–19].

Since the HSR is a hallmark of cellular response to severe heat stress, and is conserved across all domains of life, it was a surprise when Hofmann et al. [7] first reported that upregulation of HSP70 protein synthesis, the canonical indicator for HSR, was missing in the high-latitude Antarctic nototheniid *Trematomus bernacchii*. Their subsequent observation that HSP70 gene expression appeared to persist at high levels relative to its constitutive paralog (HSC71) led to the inference that this once inducible molecular chaperone had been co-opted into constitutive expression [20]. This was proposed as an adaptation to the denaturing stress from perpetual freezing marine temperatures. Absent any environmental thermal surges, selective pressure for the inducible HSR would become relaxed, allowing its loss or alteration into a constitutive response. Additional investigations into HSR in a few other Antarctic notothenioids generally corroborated the loss of the HSR in Antarctic species [21–25]. However, these studies utilized disparate heating regimes and methodologies, preventing meaningful direct comparisons between studies and species. Also, limited species sampling has provided little evolutionary context on the ancestral status of the HSR at the dawn of the Antarctic notothenioid radiation.

To enable direct comparisons across species, in this study we used the Critical Thermal Maximum (CTMax) approach [26] to provide a unified acute heat stress protocol and assessment of the stress end point. We utilized three strategically selected notothenioid lineages – the cryopelagic *P. borchgrevinki* representing the highly cold adapted and specialized red-blooded Antarctic species, *C. rastrospinosus* representing the derived hemoglobin-lacking Antarctic icefishes, and the temperate water *E. maclovinus*, the basal, non-Antarctic notothenioid and the closest sister to the Antarctic clade serving as proxy for the notothenioid ancestral state. To understand impacts across the full scope of the HSR, as well as the broader cellular responses to heat shock in the cold-specialized Antarctic notothenioids, we used RNA-Seq analyses to interrogate and compare cellular transcriptional responses to the standardized acute, severe heat stress (CTMax). Through this comparative transcriptomic investigation, we aim to understand the changes that evolution in chronic cold has wrought in Antarctic notothenioid lineages, in both the HSR and the broad suite of cellular-wide stress responses. Such understanding is particularly relevant given that Antarctic organisms are now faced with climatic changes and warming waters in the Southern Ocean.

## Results

### Sequencing, de novo assembly, and annotation of the reference transcriptomes

**Fig. 1:**
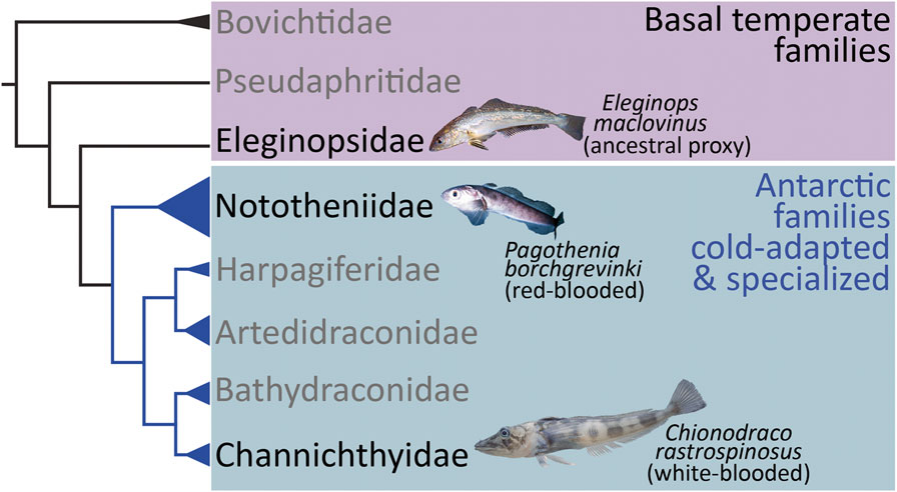
Notothenioid Phylogeny Showing the Positions of the Three Study Species. Study species are shown in black text with additional details provided. The phylogeny shows the eight notothenioid families and is divided by color between the three temperate basal families and five Antarctic families. The photo of *E. maclovinus* is courtesy of Dirk Schories

The three species were selected as representatives of phylogenetically strategic notothenioid lineages that encompass key evolutionary differences in physiology and thermal history – basal temperate, cold-adapted Antarctic red-blooded and derived Antarctic hemoglobinless conditions (Fig. 1). The two Antarctic species share superficially similar heat tolerance as determined by their CTMax values (12.1 °C ± 0.83 for *P. borchgrevinki*, and 11.6 °C ± 1.69 for *C. rastrospinosus*), both well below that of the temperate *E. maclovinus* (30.5 °C ± 0.54). To enable gene quantification of their responses to heat stress in the absence of genomic resources, and as a resource for future investigation into the genic content of these three species, we sequenced broad reference transcriptomes (Additional file 1: Table S1) for each species. These sequencing results, along with all the sequenced reads from this study have been deposited with NCBI SRA under accession number SRP098134. As summarized in Additional file 1: Table S2, the Illumina paired end 250 bp sequencing of the three species’ reference transcriptomes constructed from multi-tissue RNA pools yielded 60 million paired end reads for *E. maclovinus*, 63 million for *P. borchgrevinki*, and 67 million for *C. rastrospinosus*. Quality trimming the paired end reads to ensure high quality retained 68.8% of the *E. maclovinus* reads, 67.4% of the *P. borchgrevinki* reads, and 64.4% of *C. rastrospinosus* reads. De novo assembly and subsequent filtering resulted in transcriptomes of 157,790 contigs for *E. maclovinus*, 143,700 for *P. borchgrevinki*, and 177,320 for *C. rastrospinosus*. From these, we were able to identify 14,256 putative orthologous groups that spanned all three species, incorporating 18,697 contigs from *E. maclovinus*, 18,806 contigs from *C. rastrospinosus*, and 18,083 contigs from *P. borchgrevinki*.

**Fig. 2:**
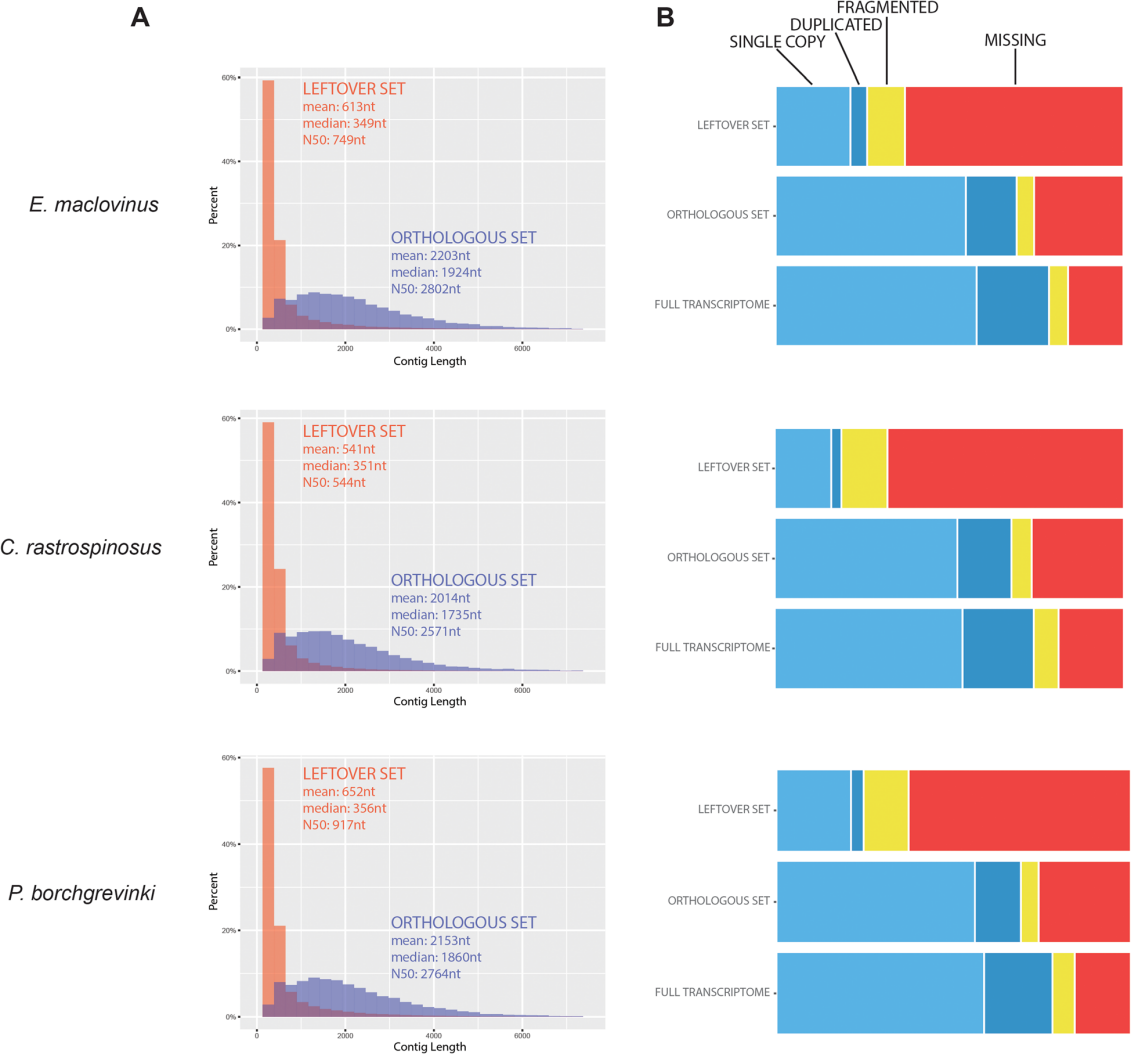
Comparison Between the Orthologous and Leftover Sets in Each Species. Column (**a**) shows the relative length distribution of contigs in the Orthologous and Leftover sets for each species, showing the orthologous set comprised predominantly longer contigs. Column (**b**) shows BUSCO evaluation results of the full assembly, orthologous set, and leftover set for each species, suggesting a preservation of much of the genic information from the full transcriptome in the orthologous set

While the orthologous sets are substantially smaller than the full assembled reference transcriptomes, the overview in Fig. 2a shows that they comprise much greater proportions of longer contigs compared to the leftover sets. Along with this length distribution, transcriptome completeness assessment using BUSCO suggests that the orthologous sets incorporated a large proportion of the genic information present in the original transcriptomes (Fig. 2b). Together, these support that the orthologous sets comprise richer information portions of each species’ respective assembly, and thus are most relevant for downstream analysis. BUSCO results further demonstrate that the species’ full transcriptome assembly and their final orthologous sets, were comparable in genic content and completeness in all three species (Fig. 2b).

### Transcript mapping of CTMax stressed fish

Investigation into the impact of evolution in chronic cold on the acute response to heat stress was based on expression in gill, taken from control and specimens sampled 2 h after being warmed to their CTMax. Gill library and mapping results are summarized in Additional file 1: Table S3 (read counts) and Additional file 1: Table S4 (mapping percentages). The majority (average of 52.6 to 57.3%) of quality filtered sequence reads from gill RNA of each species mapped to the orthologous set of the respective species (Additional file 1: Table S4). While there was some difference in mapping efficiency between species, it is comparable between control and heat stressed specimens within species. The fact that the orthologous sets do not account for all of the genic content of the full transcriptomes, as indicated by approximately one-third of the quality filtered reads mapping to the leftover sets, underscores the inherent challenges of ortholog detection among species when their reference genomes are not available. These include the naturally fragmentary and incomplete nature of de novo assembled transcriptomes, differing sets of expressed genes between species, and in teleost fishes the additional ambiguity that arises from their ancestral genome duplication and subsequent differential loss. Despite this, as our downstream analysis demonstrates, a clear transcriptional response to acute heat stress is evident even among this restricted set.

### Patterns in gene expression and differential gene expression

**Fig. 3:**
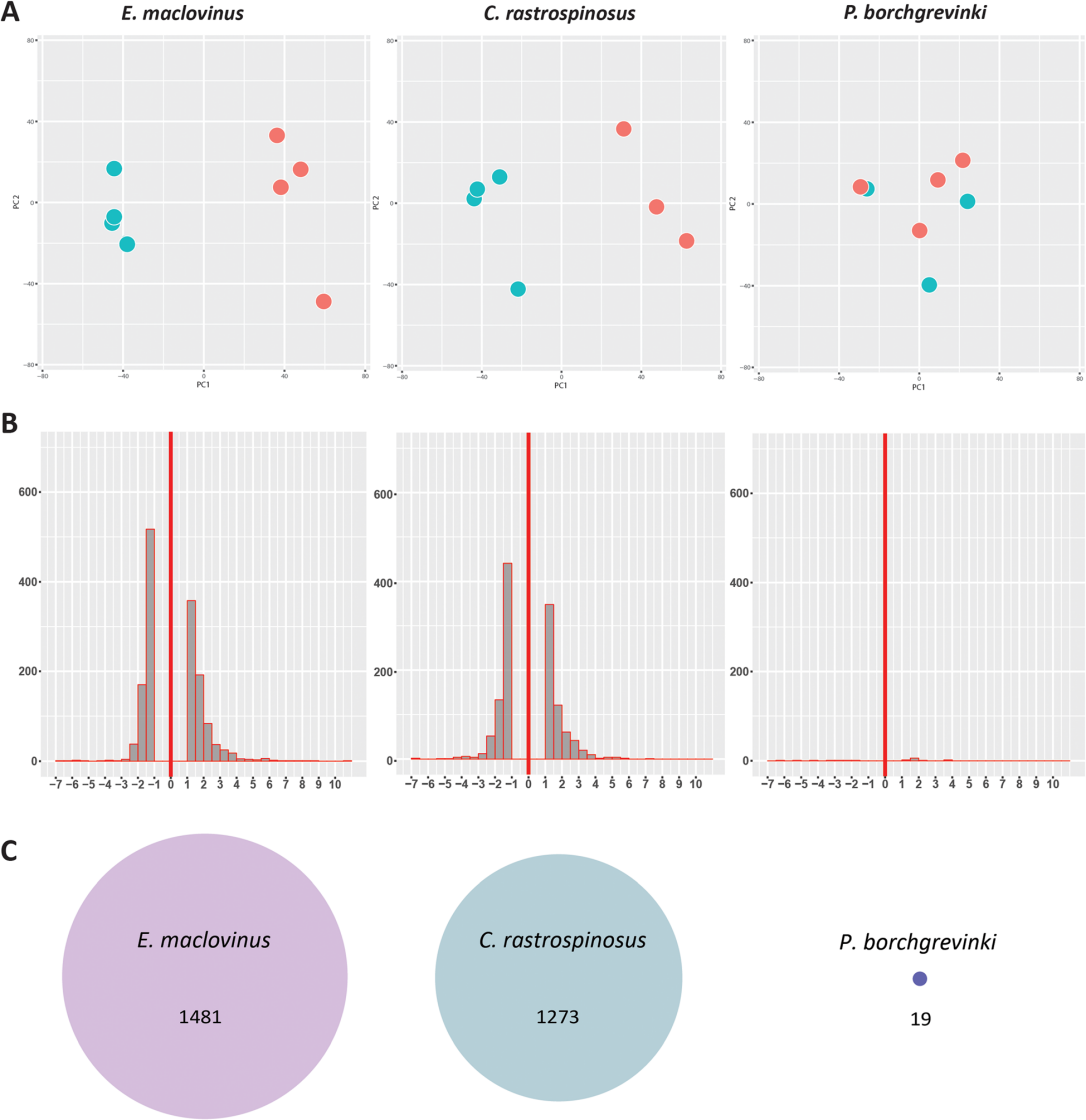
Trends in Species’ Gene Expression. **a** PCA of gene expression profiles for the three notothenioid species; blue circles represent ambient temperature controls and red circles represent individuals heated to CTMax followed by 2 h of recovery time in ambient water. Clear separation of expression profiles between controls and heat stressed individuals was found for *E. maclovinus* and *C. rastrospinosus* but not for *P. borchgrevinki*. One control sample was removed from *P. borchgrevinki* and one heat shocked sample from *C. rastrospinosus* both as outliers. **b** Histograms showing changing expression of differentially expressed genes in all three species. Y-axis is log_2_FC and X-axis is the number of responding genes (**c**) Scaled schematics comparing the magnitude (in numbers) of differentially expressed genes detected in the three species

Initial evaluations of broad trends in gene expression revealed clear impact of severe heat stress in two of the three notothenioids (Fig. 3). Both the basal, temperate *E. maclovinus* and the Antarctic icefish *C. rastrospinosus* displayed different overall gene expression profiles between the ambient controls and heated individuals (Fig. 3a). These mounted similarly robust transcriptional response to the CTMax heat stress (Fig. 3b, c), with the temperate *E. maclovinus* showing a larger response as measured by the number of differentially expressed genes (1,481 versus 1,273 in *C. rastrospinosus*) (Fig. 3c). In stark contrast, *P. borchgrevinki* showed no clear differentiation in gene expression between control and heat stressed specimens (Fig. 3a), and correspondingly very few (19) differentially expressed genes were detected (Fig. 3b, c).

**Fig. 4:**
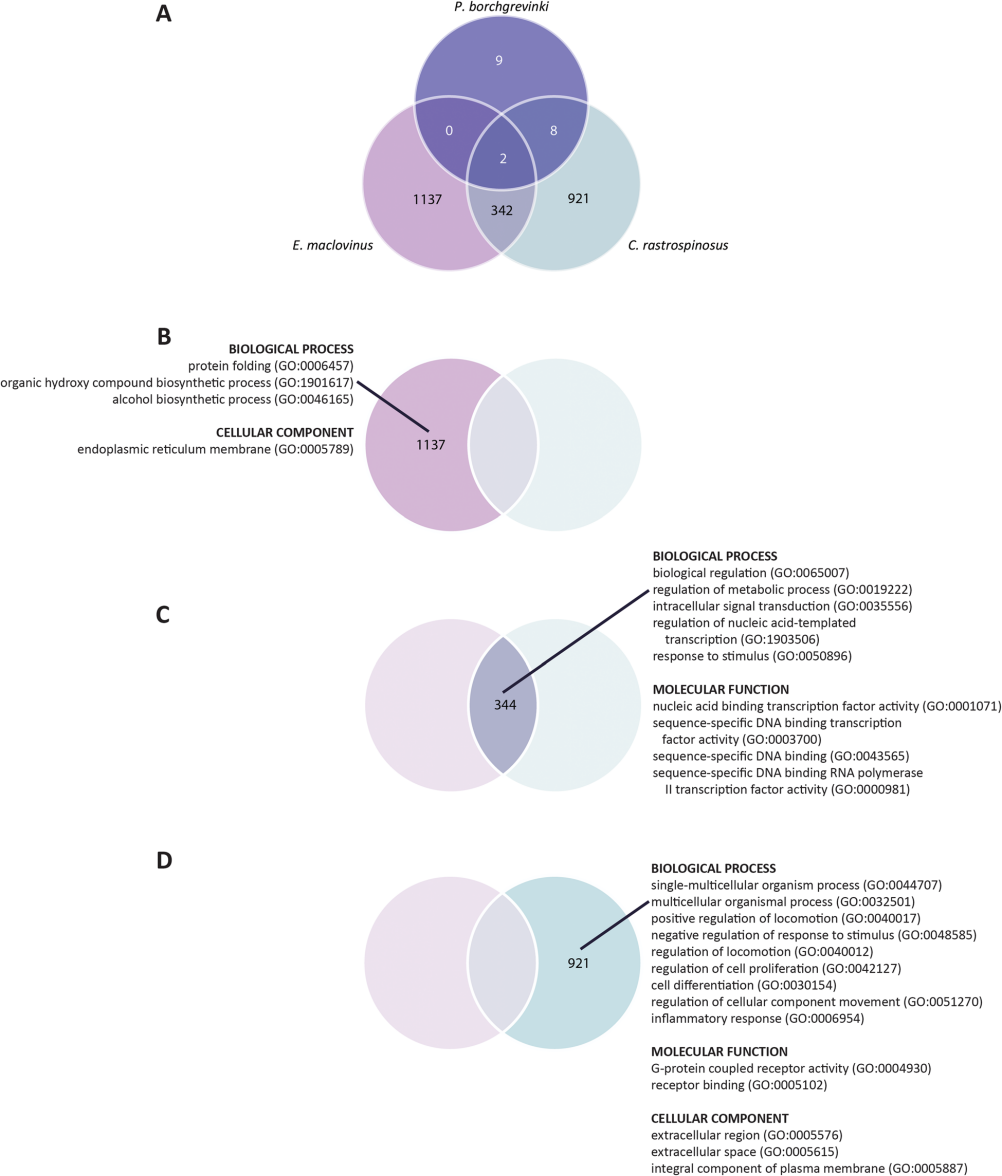
Shared and Species Specific Enrichment Analysis Results. The Venn diagram for the three examined species and significantly enriched terms according to each segment. GO lists in the figure were summarized using REVIGO in order to remove redundancy

Comparison of the suite of responding genes revealed distinctive underlying differences between the responses of *E. maclovinus* and *C. rastrospinosus* to heat, in that the majority of differentially expressed genes were species specific, 1137 of 1479 (76.9%) and 921 of 1263 (72.9%) respectively (Fig. 4). The smaller, shared set of 344 responding genes showed a generally consistent response in the direction and magnitude of change in expression (correlation coefficient 0.74). Only 18 of these shared genes showed an opposite response in the two species, and they were themselves functionally diverse in nature. Given the paucity of differentially expressed genes in *P. borchgrevinki*, only two genes were identified as shared with the other two species (Fig. 4a). Interestingly, these two included the transcription factor *c-Fos* that is characteristically associated with stress response, as well as a ubiquitin E3 ligase putatively identified as E3 ubiquitin-protein ligase RNF182.

### GO enrichment

To put these differentially expressed genes in biological context we tested for functional enrichment by GO enrichment analysis. We identified five enriched GO terms for the differentially expressed genes specific to *E. maclovinus* (Additional file 1: Table S5), 22 enriched GO terms for the differentially expressed genes shared by *E. maclovinus* and *C. rastrospinosus* (Additional file 1: Table S6), and 23 enriched GO terms for the differentially expressed genes specific to *C. rastrospinosus* (Additional file 1: Table S7). The shared and species-specific responses in enriched GO terms were summarized with the help of REVIGO [27] to remove redundancy in GO terms and presented in Fig. 4. For reference, the global responses (read mapping to full transcriptomes) of *E. maclovinus* and *C. rastrospinosus* responses were similarly tested for enrichment, with 13 enriched GO terms found for *E. maclovinus* (Additional file 1: Table S8) and 81 enriched GO terms for *C. rastrospinosus* (Additional file 1: Table S9). Enrichment results themselves were functionally consistent when comparing species’ global responses and the combined species specific and shared responses. The small number of differentially expressed genes in *P. borchgrevinki* were not enriched for any GO terms.

### Expression of molecular chaperones

**Fig. 5:**
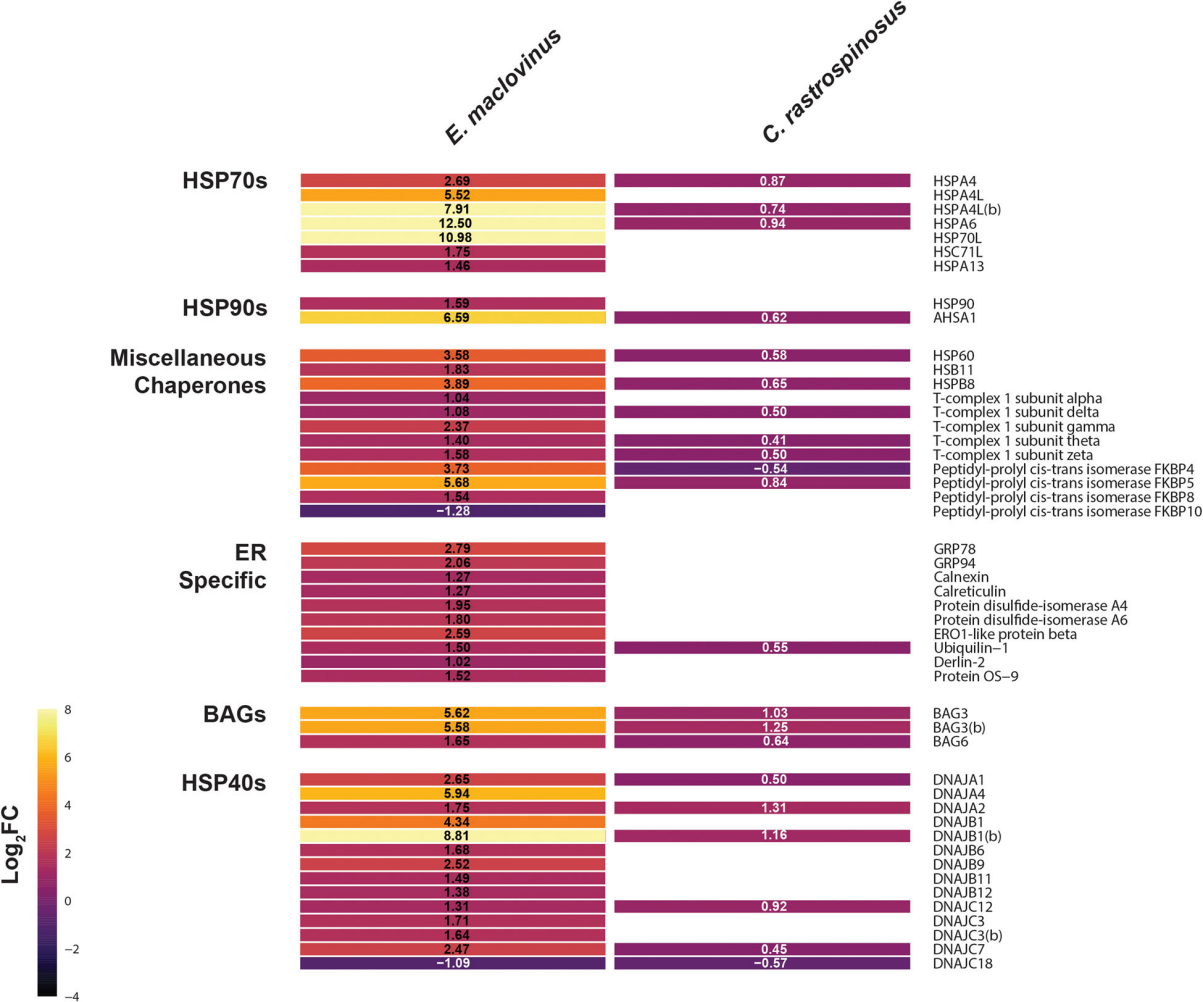
Heatmap of Identified Molecular Chaperones and Related Genes. Demonstration of the substantially differing expression of genes associated with the HSR in *E. maclovinus* and *C. rastrospinosus*. Only genes significant at a 0.1 FDR adjusted *p* value are shown. Each cell shows the estimated log_2_FC and FDR adjusted *p* value in parentheses. As no genes associated with the HSR were found significantly different in *P. borchgrevinki* even at this relaxed threshold it is excluded from this figure

Given the apparent loss of the HSR, the responses of molecular chaperones were of particular interest in the Antarctic notothenioids. Thus, we directly examined the changing expressions of molecular chaperones and related genes using a relaxed thresholds for significance (FDR 0.1) in order to detect any vestige of a response in the Antarctic species (Fig. 5). We found that where differential expressions of molecular chaperones were detected in *C. rastrospinosus*, they were greatly diminished compared to those in the basal, temperate *E. maclovinus* (Fig. 5). No vestigial response could be detected in *P. borchgrevinki* even at the reduced threshold for significance.

## Discussion

Evolution in the chronically freezing Southern Ocean has had pervasive biological impacts on the Antarctic notothenioid fishes, including changes in the activity and regulation of gene expression. At its native water temperatures, the giant Antarctic toothfish *Dissostichus mawsoni* shows signs of adaptive change in its pattern of gene expression that act to mitigate the cellular challenges from the unrelenting cold of the Southern Ocean [28]. These include augmented expression of genes participating in protein biosynthesis, protein folding and degradation, lipid metabolism, anti-oxidation, and anti-apoptosis pathways. Notothenioid specialization to cold is also exemplified by a radical deviation from the expected changes in gene expression that heat stress typically induces in other organisms. In particular, these fishes share an apparent inability to induce heat shock protein expression [22, 25], normally a key transcriptional response to severe heat stress. Here, using a standardized heat stress protocol and stress end point allowed us to meaningfully compare transcriptional responses among the three select notothenioid lineages, and evaluate and trace the impact of evolution in chronic cold on the heat stress response. We found that the transcriptional capacity to respond to acute heat stress is not uniformly lost or diminished across Antarctic notothenioid species as commonly noted to-date, but can differ drastically between species as observed in the two Antarctic species in this study.

### The *E. maclovinus* specific response

As the nearest extant temperate notothenioid to diverge before the Antarctic radiation [29], the *E. maclovinus* response to acute heat stress provides a window into the ancestral state of the notothenioid response prior to the onset of freezing conditions and chronic cold. By comparison to the responses of Antarctic species, identifying what is specific to *E. maclovinus* helps to isolate the functional responses that have been altered or lost with Antarctic notothenioid evolution in the chronically freezing Southern Ocean. Notably, the *E. maclovinus* specific response contained the conserved primary transcriptional responses to denaturing stress from heat, observed across all domains of life. Prominent within this response was the strong induction of genes responsible for protein folding (GO:0006457) (Fig. 4b, Additional file 1: Table S5), including many constituents of the HSR. As shown in Fig. 5, particularly strong induction was seen among the various classes of molecular chaperones and supporting genes including members of the *HSP70* chaperone family, *HSP90*, various co-chaperones for *HSP70* and *HSP90*, as well as in the *HSP70* mediator *BAG3* (Fig. 5). In clear contrast, the response of the same classes of genes in *C. rastrospinosus* is very limited, in both the small number of responding genes and the diminutive level of their induction. Further muted to an extreme in *P. borchgrevinki*, none of the chaperones were found to be induced even under the relaxed thresholds used for this comparison.

The enrichment analyses also identified the endoplasmic reticulum (ER) as a particular site of transcriptional activity in *E. maclovinus* following acute heat stress (GO:0005789) (Fig. 4b; Additional file 1: Table S5). The denaturing impact of severe heat would be expected to impact the protein pool within the ER as much as the cytosolic pool, leading to ER stress from the accumulation of denatured, unfolded, and misfolded proteins within the ER lumen. Evidence for transcriptional response to such ER stress, and activation of the UPR, include induction of a variety of ER-specific chaperones such GRP78, Endoplasmin, Calreticulin, the oxidoreductase ERO1B, along with changing expression of two protein disulfide isomerases (Fig. 5). Besides the ER chaperones, there were signs of a more widespread response targeting ER functions, including induction of numerous genes involved in ER to Golgi transport as well as lipid and cholesterol biosynthesis. As with the HSR, the Antarctic species showed a response that was either incomplete and muted as in *C. rastrospinosus* (Fig. 5), or wholly absent as in *P. borchgrevinki*.

The HSR and UPR share cellular machinery in restoring proteostasis (protein homeostasis) and appear interconnected by crosstalk in their respective regulatory networks [30, 31]. However, the exact nature of this interaction during periods of severe proteolytic stress is still poorly understood [32]. What we have observed here is evidence for induction of many of the effectors of the UPR alongside those of the HSR in *E. maclovinus*, though not in the Antarctic species (Fig. 5).

The evolutionary status of UPR to ER stress is poorly understood. Recent work associated with investigation of the Antarctic *Notothenia coriiceps* genome noted a lack of transcriptional signals relevant to ER stress following one and 2 days of mild 4 °C warming [33], while earlier work by Clark et al. [23] found repression of GRP78, a key effector of the UPR, in *Harpagifer antarcticus* following the acute heat shock of 2 h at 10 and 15 °C. These prior results, along with our current finding suggest that the UPR, like the HSR, may not activate in Antarctic species, at least in response to heat stress. There is a real need for further experimental investigation into the native ER proteostasis activity in ambient cold, and how it is affected by added stress from heat.

### The shared *E. maclovinus* and *C. rastrospinosus* response

**Fig. 6:**
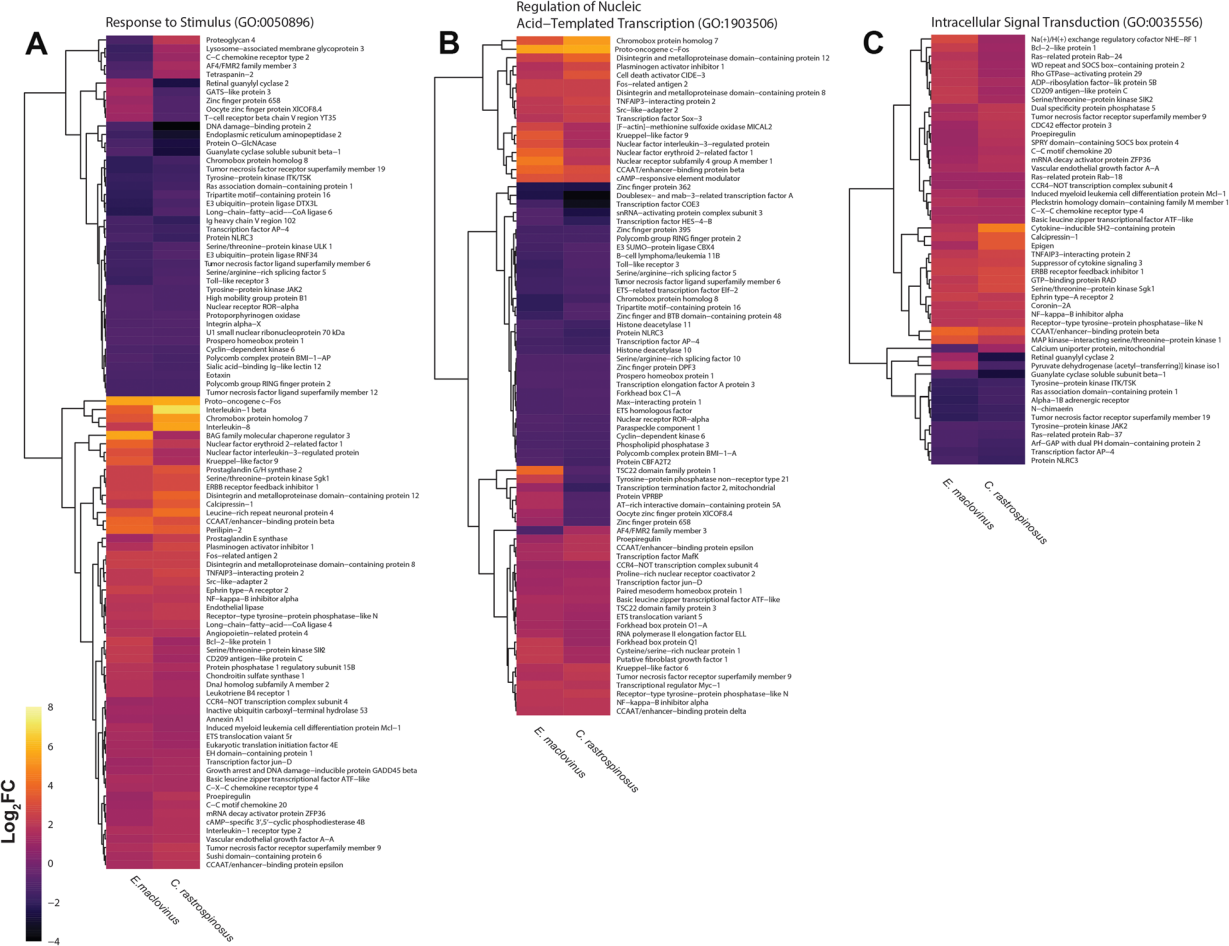
Comparison of Transcriptional Activity of Differentially Expressed Genes in Key Pathways of the Shared Response. Heat maps for genes differentially expressed in both *E. maclovinus* and *C. rastrospinosus* following heat stress, for genes that belong to the Response to Stimulus (GO:0050896), Regulation of Nucleic Acid-Templated Transcription (GO:1903506), and Intracellular Signal Transduction (GO:0035556)

Beyond the iconic molecular chaperones of the HSR, environmental stresses instigate rapid changes in transcriptional activities in other cellular-wide gene networks. Despite a largely quiescent HSR and UPR, the Antarctic icefish *C. rastrospinosus* exhibited a set of heat activated transcriptional responses common with *E. maclovinus* (Fig. 4c; Additional file 1: Table S6). Of these, the enriched response to stress and cellular stimulus (GO:0050896) suggest the broader cellular stress response remains engaged by acute heat stress in this species. This includes the shared induction of a diverse group of genes commonly triggered by cellular and environmental stresses (Fig. 6a), among which are genes with broad roles in the stress response such as *SGK1* (serine/threonine-protein kinase Sgk1) and *SIK2* (salt inducible kinase 2), genes that respond to DNA damage such as *GADD45B* (growth arrest and DNA damage inducible beta) and *DDIT4L* (DNA damage inducible transcript 4 like), genes associated with cell death or cell survival such as *B2CL1* (Bcl-2-like protein 1), as well as the mediators of the inflammation response, Interleukin-1 beta and Interleukin-8.

More broadly yet, the heat stress appears to trigger wide-ranging reorganization of biological and metabolic regulatory processes (GO:0065007, GO:0035556). Central to this was changing expression of numerous stress responsive transcription factors (GO:1903506, GO:0001071, GO:0003700, GO:0043565, GO:0000981) (Fig. 6b), which are frequently observed in the transcriptional response to diverse forms of stresses [34]. Particularly prominent here for their biological importance, and as biomarkers of cellular and organismal stress [35, 36], were inductions of members of the Immediate-early transcription factor families *Jun, Fos,* and *Maf* (Fig. 6b). Also induced were transcription factors known to respond quickly to cellular stresses (*CCAAT/EBP β, δ* and *ε*, *KLF6*, and *KLF9*), or have relevant roles in stress response (*CREM, NFIL3, NFE2L1, NR4A1, FOXQ1*). Of these, upregulation of the CCAAT/enhancer-binding proteins (*CCAAT/EBP*) has been inferred to be of particular importance in a heat stress study of the high-latitude nototheniid *Trematomus bernacchii* [37].

Cell signaling pathways are known to play critical roles in activating and mediating cellular responses to environmental stress. As such, signaling activities themselves are tightly regulated to ensure an organized cellular response [14]. Accordingly, they appear to be a second key target in the shared transcriptional response (GO:0035556; Fig. 4c) between *E. maclovinus* and *C. rastrospinosus*. This regulation was found to impact several discrete signaling pathways (Fig. 6c), with specific impacts noted on NFKB activity (by *IKBA*, *TNIP2*), MAPK signaling activity (by *DUSP5, MKNK1*), JNK signaling (by *TNFRSF19*), and cytokine signaling (by *CISH, SOCS3*). Additionally, the broader shared response also included induction of several genes regulating G-protein activity (by *GPSM1, RGS2, RGS4, RGS13*) and *TRIB2* (Tribbles 2) - a mediator of MAPK signaling, which was one of the most strongly induced genes in *C. rastrospinosus* (4.3 log_2_FC).

### The *C. rastrospinosus* specific response

The great majority of heat responding genes in *C. rastrospinosus* (921/1263; 72.9%) participated in a large species-specific transcriptional response. The first investigation of the acute stress response in an Antarctic icefish, these suggest that two hours after warming to their CTMax heat stress had instead triggered widespread activation of the inflammation response, unlike the HSR which predominated in the *E. maclovinus* specific response (Fig. 4d; Additional file 1: Table S7). Inflammation itself can be induced by cellular perturbations caused by internal or external stimuli, and is considered to be the end of the spectrum of mechanisms that defend and restore homeostasis [38]. It is therefore possible that inflammation pathways are more quickly dominant in the Antarctic fish where the HSR is not available to ameliorate cellular stress at the front line. While we did see the induction of several key mediators of inflammation in the shared response, in particular induction of the pro-inflammatory agents interleukin 1 and 8, the organized response was only fully observed in *C. rastrospinosus* (GO:0006954; Fig. 4d), where it drove enrichment of many of the observed GO categories.

Beyond the interleukins, direct indicators of inflammation activity in *C. rastrospinosus* include upregulation of two Death-associated protein kinases (− 2 and − 3), receptors associated with inflammation response (*LTB4R, PTAFR*), and a number of genes regulating inflammation response or as part of immune activity (*THBS1, TBK1, TNFRSF14, C4B, CCL20, CXCL6, PSTPIP1*). The differential expression of these and other genes tied to the inflammation response further drove enrichment of many of the observed GO terms seen in the *C. rastrospinosus* specific response (Fig. 4d), including GO terms describing broad organismal and extracellular activity (GO:0044707, GO:0032501, GO:0005576, GO:0005615), impacting cell differentiation and proliferation (GO:0030154, GO:0042127), regulating cellular component movement (GO:0051270, GO:0040017, GO:0040012, GO:0005887), as well as many acting through receptor activity (GO:0005102, GO:0004930). This broader suite of genes included the induction of a number of matrix metalloproteinases (MMP2, MMP14, MMP19) which act to regulate the inflammation response and associated downstream genes related to integrity of the extracellular matrix as well as a number of growth factors [39, 40]. Of the latter, these included the fibroblast growth factors *FGF2* and *FGF4*, betacellulin (*BTC*), heparin-binding EGF-like growth factor (*HB-EGF*), as well as vascular endothelial growth factor C (*VEGF-C*).

Within this inflammation response we also saw the induction of several genes that would act to increase blood flow. These include strong induction of inflammatory agent and vasodilator *ADM* (adrenomedullin), induction of the vasodilatory receptor and apoptosis mediator *AGTR2* (angiotensin II receptor type 2), as well as changing expressions of several genes that would act to limit clotting (*PLAU, PLEK, GP5, GP1BA*). Outside the inflammation response, these may serve an additional role in the icefishes where it could also help to clear tissue oxidative damages which appears to be a particular challenge for these hemoglobin lacking species following heat stress [41]. Finally, while *C. rastrospinosus* shows clear signs that stress signaling pathways have been engaged, their activities appear to be more rapidly attenuated (GO:0048585; Fig. 4d). This suggests that the response might naturally progress more rapidly in this species, or it may be under suppression in the absence of the heat shock proteins, the primary effectors of the cellular response.

### The *P. borchgrevinki* response

Compared to the other two species, *P. borchgrevinki* was extraordinary for the near lack of observed transcriptional response two-hours after exposure to severe heat stress, with only 19 detectable differentially expressed genes (Fig. 4a). This disparate response was observed even though all three fishes were brought to the same physiological endpoint in their CTMax which elicited robust transcriptional responses in the other two species. Among the few genes that did respond were several stress responsive transcription factors suggesting some residual transcriptional sensitivity to acute heat stress and including *Jun-B* (1.6 log_2_FC), *c-Fos* (3.5 log_2_FC), *IER2* (2.1 log_2_FC), as well as *EGR1* (1.2 log_2_FC).

The “missing” response in *P. borchgrevinki* is peculiar. To ascertain whether this lack of response was due to limits of detection in our statistical analysis, we rigorously tested additional possible avenues through which this species-specific response may have been obscured. This included investigation into possible batch effects [42] and a broad reanalysis using limma with Voom [43], which both validated the lack of response (Additional file 2). Additionally, we tested for impacts that might result from the stringency of the minimum count thresholds (Additional file 3) as well as the FDR adjusted *p* value threshold (Additional file 4), neither of which resulted in any meaningful change in the muted response in *P. borchgrevinki* observed in our initial analysis. These results support a biological basis for the “missing” response in this species rather than a failure of analyses to detect.

While past work looking at moderate warming over extended periods has found larger transcriptional responses [21, 44], the acute response, especially to severe temperatures, has not previously been investigated in this species and which appears substantially more muted in this study. Warming to the CTMax itself has previously proven capable of inducing transcriptional responses to acute heat stress in fishes [45], as also demonstrated here in the two other examined species. The muted response seen in *P. borchgrevinki* may reflect exceptional insensitivity to heat such that none of the responding pathways to acute heat stress are invoked, as these differ from the homeostatic responses profiled in prior investigations of this species. Heat stress in ectotherms normally triggers a cellular response that increases with temperature until an upper physiological limit is reached [46]. At this point the physiological state becomes so perturbed that further increases in temperature limits transcription of even stress related gene. It is therefore a possibility that the high-latitude notothenioid species, inhabitants of particularly cold stable water temperatures, may now only be able to transcriptionally respond to relatively mild heat stresses and that warming to the CTMax would now represent an excessive heat stress in these fishes.

The cellular response itself is a kinetic process and one reasonable testable hypothesis is that the time course of response to severe heat in *P. borchgrevinki* is drastically different from the two other investigated notothenioid species. Such a drastically differing rate of response compared to the other two studied species would itself represent an interesting deviation from the expected time course of the heat stress response. Thus, further investigations are needed to determine whether the extraordinary transcriptional inactivity observed in *P. borchgrevinki* is due to an unusual disparate rate of response, lesions limiting the activation of the broader transcriptional responses to acute stresses, or a greatly reduced cellular capacity to respond to temperatures above 4 °C.

## Conclusion

As the most relevant ancestral proxy for Antarctic notothenioids, the heat induced transcriptional response of the basal temperate *E. maclovinus* provides a window into its status prior to the influence of chronic freezing polar marine conditions. The robust HSR and UPR of *E. maclovinus* to heat stress clearly indicate they were intact in the most recent common ancestor of the Antarctic radiation, confirming these responses as a plesiomorphy in Notothenioidei. Additionally, with an organized response to denatured proteins specific to just *E. maclovinus*, this study shows that both the classic HSR and the ER specific UPR appear missing in response to severe heat stress among the Antarctic notothenioids.

The regulatory basis of these losses in the Antarctic notothenioids remains to be ascertained. Given that the loss of the HSR has consistently been seen with a lack of induction across all of the relevant molecular chaperone genes, it suggests the cause is likely a central lesion upstream in the pathway, rather than in the regulatory elements of individual genes. The cause of inactivity in the UPR is similarly puzzling given that the HSR and UPR are under differing transcriptional control and the need for the UPR should be greater with the inactivity of the HSR. Why the buildup of denatured macromolecules is somehow reduced or not detected within these pathways remains unknown. Detection of such protein damage in these cases relies on discrete signal transduction pathways whose activity is not directly assessed in transcriptional analyses and thus it remains to be determined whether these signal transduction pathways have been activated by heat stress. It further seems unlikely that the response to ER stress itself would be wholly lost in these fishes given its importance in day to day ER function in protein proteostasis.

Beyond the direct responses to denaturing stress, the three species differed notably in the magnitude and content of their respective transcriptional responses. It was surprising to find that the largest difference in heat stress response was not between the temperate and Antarctic species, but between the two Antarctic species. The near-nonexistent response in *P. borchgrevinki* was unexpected and presently without clear reason. The cause of this greatly muted response, and whether it is unique to *P. borchgrevinki* or occurs in other notothenioid species, can only be ascertained by further investigations.

Prior studies have shown that selective pressure will act on the stress response leading species to differ in the magnitude and timing of their responses to stress. Investigating four species of marine snails of the genus Tegula occupying distinct thermal niches, Tomanek and Somero [46] found evidence for genetically fixed differences not only in the temperature at which the HSR is triggered and peaks but also the magnitude of the peak response, reflecting the separate evolutionary histories of these species. They similarly found that the rate of progression through a stress response appears to differ substantially between species [47], which could lead to observations of large differences in a response when compared at only a single point along the time course of a response. Presumably such differences in the HSR are reflective of the broader cellular stress response which we would expect to also differ between species in sensitivity and responsiveness based on the environmental challenges faced through their respective evolutionary histories.

Transcriptional responses may vary with life stage [48] and it is currently unknown whether the response observed in the juvenile *E. maclovinus* used in this experiment differ from that of adult specimens. Similarly, specimens of *P. borchgrevinki* and *C. rastrospinosus* used in this experiment were wild-caught and the responses seen in these species could be influenced by year class effects and other epigenetic influences. These are implicit challenges present in the study of many non-model organisms and though their impacts are outside our capacity to isolate in the current study, we will likely be better able to investigate them as more temperate and Antarctic notothenioid genomes become available.

Given the rapid warming seen in parts of the Antarctic [49–51]**,** the limited heat tolerance seen in Antarctic notothenioid fishes along with their relatively limited capacity for phenotypic plasticity would leave them particularly vulnerable by anticipated future increases in water temperature. The evolution of the Antarctic notothenioid fishes in the cold-stable water temperatures of the Southern Ocean would have relaxed selective pressure on classic transcriptional responses to heat stress. Understanding the nature and extent of these losses may help inform us of their capacity to adapt to rising temperatures and to survive future warming. The loss of the HSR on its own is known to prevent the development of cellular thermotolerance [52], and where a wider portion of the cellular stress response may have been removed or silent, such as in *P. borchgrevinki*, this may leave the underlying network of genes and regulatory elements that would normally enable its activity, at risk of degradation and loss. If such losses have occurred throughout the mechanisms of these responses in some or all of the Antarctic notothenioids, then this may greatly limit their capacity for future adaptive change to warming conditions.

## Methods

### Collection and experimental heat stress

Specimens of *P. borchgrevinki* were collected during the austral summer months of October and November 2012 by hook and line through holes drilled through the sea ice of McMurdo Sound. The fish were then transported back in aerated coolers to the Crary research lab at McMurdo Station where they were held in flow through aquaria at ambient water temperatures (≤ − 1.6 °C). Specimens of *C. rastrospinosus* were collected by trawl from aboard the R/V L.M. Gould at sites along the Western Antarctic Peninsula during the austral winter of 2014. These were transported in the ship flow through tanks to Palmer Station where they were held in flow-through aquaria at ambient water temperatures (≤ − 1.0 °C). Specimens of *E. maclovinus* were acquired during the austral winter of 2014 using a small seine in the near shore water of Valdivia, Chile. This cohort happened to be juvenile specimens. They were held at ambient temperature (~ 15 °C) in flow through aquaria at the Laboratorio Costero de Recursos Acuáticos Calfuco of the Universidad Austral de Chile. Each species was allowed a week or more to recover from collection stress in its respective flow through aquarium before the heat stress experiment. Collection, care and experimentation were carried out in compliance with Protocol # 12123 approved by the University of Illinois IACUC (Institutional Animal Care and Use Committee) and Permit # 11/10 issued by the Universidad Austral de Chile Comité Bioética on Uso de Animales en la Investigación.

For acute heat stress, specimens were heated to their CTMax temperature as described in Bilyk and DeVries [4]. This standardized protocol for applying acute severe heat stress and assessing common stress end point enabled direct comparisons across species despite the vastly different native thermal habitats and distinct CTMax values between the temperate *E. maclovinus* and the two Antarctic species. Fish were put in 30 or 40 gal glass aquaria of ambient temperature water. With vigorous aeration in place, the water was heated with one or two 300 W titanium aquarium heaters to achieve a heating rate of 0.3 °C per minute until a persistent loss of equilibrium was observed in the fish, i.e. unable to right itself. Upon reaching this CTMax end point, fish were immediately returned to their ambient water temperatures to recover and allow tissue transcriptional responses to manifest. All fish survived this heat treatment and specimens were sacrificed at both two and 4 h of recovery time post-CTMax. In accordance with UIUC IACUC Protocol #12123 these fish were anesthetized in MS222 (tricain methane sulfonate) at 1 g/15 L seawater then quickly euthanized by severing the spinal cord using a scalpel. Tissues were then quickly dissected and submerged in a large excess (10-20× volume) of − 80 °C pre-chilled 90% molecular biology grade ethanol to preserve RNA. The ethanol was refreshed twice or three times within the following few hours to ensure inactivation of nucleases by thorough dehydration. Fully ethanol-equilibrated tissue samples were stored in -20 °C until they were shipped on dry ice back to the University of Illinois, Urbana Champaign where RNA extractions were carried out.

### RNA extraction, library preparation, and sequencing

All RNA extractions were carried out using a Bullet Blender^R^ homogenizer with Zirconium 0.5 mm beads (Next Advance Inc., Averill Park, NY) and the Ultraspec RNA isolation system (Biotecx Laboratories Inc., Houston, TX) according to manufacturer’s instructions. The resulting RNA pellets were dissolved in 50–100 μL of 0.5× Tris-EDTA buffer (TE) (5 mM Tris–HCl, 0.5 mM EDTA, pH 8.0), then treated with DNase I (New England Biolabs Inc., Ipswich, MA) at 37 °C for 20 min to degrade potential contaminating genomic DNA, followed by cleaning the treated RNA samples using ZYMO RNA Clean & Concentrator-25 columns (Zymo Research Inc., Irvine, CA). Initial assessment of RNA integrity was done using the Epoch Take 3 microplate spectrophotometer (BioTek Instruments, Winooski, VT), measuring OD260/280 ratios and concentration, then by visualizing RNA quality of 1 μg from each sample electrophoresed on a 1% denaturing formaldehyde agarose gel. Final sample concentration was measured with a Qubit 2.0 Fluorometer (ThermoFisher Scientific Inc.), and RNA quality in RIN (RNA Integrity Number) determined using an Agilent 2100 Bioanalyzer (Agilent Technologies Inc., Santa Clara, CA).

Two sets of sequencing libraries were prepared for each species. The first was a single library encompassing pooled RNA from multiple tissues and experimental groups to generate a reference transcriptome specific to each species. Annotated reference transcriptomes were needed in the absence of sequenced genomes for these three species in order to map transcript reads from experimental individuals and quantifying their expression levels. The second consists of eight individual libraries of gill RNA per species, from four ambient control and four heat stressed individuals sampled two-hours after warming to their CTMax, to measure the transcriptional impact of acute heat stress on this representative tissue.

For the reference transcriptomes, a summary of the tissues, treatments, and specimens incorporated into each species pool, along with their respective RIN values, is presented in Additional file 1: Table S1. For each species, a final RNA pool was constructed from equal quantities of individual tissue RNA pools. There were 10 tissue RNA pools: liver, gill, brain, spleen, muscle, stomach, lens, eye cup, intestine, and head kidney, to capture organismal transcription response broadly. Where both genders were available, each tissue pool comprised equal quantities of RNA from one representative male and female specimen from the control, two-hour, and four-hour post CTMax recovery groups. This allowed us to capture gene expression profiles under native and heat stressed conditions, along with any gender specific differences in gene expression. Gender determinations were not possible for *E. maclovinus* as they were sexually immature juveniles, but where possible we included two juvenile specimens per treatment as a way of capturing intra-species differences in gene expression. An indexed sequencing library for each species’ final RNA pool was prepared by the Roy J. Carver Biotechnology Center using Illumina’s TruSeq Stranded RNAseq Sample Preparation kit, and normalized with duplex-specific nuclease (Evrogen). The three libraries were then quantified by qPCR, pooled in equimolar concentration, and sequenced on one lane of Illumina HiSeq2500 for 251 cycles from both ends.

To investigate broad cellular-wide gene expressions in response to the severe heat stress, gill was selected because it is an external tissue directly exposed to the heated water. Also, gill has been used in other recent RNA-Seq investigations of notothenioid fishes [45, 53] facilitating future cross species comparisons. Gill RNA sample of four individuals from the CTMax plus two-hour recovery group were used to represent the heat stress state. Results in our companion study show the strongest expression of HSP70 in *E. maclovinus* at this time point relative to other recovery times, and suggesting a generally greater transcriptional response can be gleaned (Cheng in prep). Following quality verification, the RNA samples were submitted to the Roy J. Carver Biotechnology Center for indexed library construction using Illumina’s TruSeq Stranded RNAseq Sample Preparation kit. A total of 24 indexed libraries (eight per species) were quantified by qPCR, pooled in equimolar concentration, and sequenced on three lanes on Illumina HiSeq2500 for 161 cycles from one end.

### Assembly and annotation of the reference transcriptomes

A schematic of the full bioinformatic workflow is presented in Additional file 5: Figure S1. Prior to assembly, the paired end reads for each species’ reference transcriptome were quality filtered using Trimmomatic v0.32 (Bolger, et al. 2014) with settings as described in Additional file 6. The three reference transcriptomes were then assembled de novo from the cleaned reads using Trinity v2.2.0, incorporating strand information and using digital normalization [54]. The assembled transcriptomes were filtered to remove contigs with poor support based on the original read counts and using an FPKM threshold of one. To enable cross species comparisons, we identified putative orthologs across the reference transcriptomes of the three species using Inparanoid and Multiparanoid [55, 56] based on predicted protein coding sequences from TransDecoder v3.0.1. We retained only those orthologous groupings that included representatives from all three species. Where multiple protein coding sequences were predicted for a single contig and their ortholog assignments were not in agreement, these were split into their respective Coding Domain Sequence (CDS) set for downstream analyses.

After ortholog assignment, the transcriptomes were divided into orthologous and leftover sets of contigs, which were both annotated using Trinotate v3.0.1 in order to provide functional context. Finally, the relative completeness of these transcriptomes was evaluated using BUSCO v2 [57], a reference-based tool that estimates completeness based on representation and integrity across a set of near-universal single-copy orthologs. This was used to compare assemblies between species, as well as to assess the relative genic content of each species’ orthologous set compared to the leftover set of contigs.

### Analyzing changes in gill gene expression

To assess changing gene expression following heat stress, we enumerated transcript frequencies for each specimen’s sequenced gill libraries. Gill sequence reads in each library were first quality cleaned using Trimmomatic v0.32 (Additional file 6), then mapped to the orthologous set of their respective species’ reference transcriptomes using RSEM and Bowtie2 [58] to quantify read frequencies. At this point, one control *P. borchgrevinki* sample was identified as anomalous due to extremely low mapping efficiency (see Additional file 1: Table S4) and it was removed, reducing the total number of gill libraries for further analyses by one, to 23. Transcript read counts were then converted to gene counts based on ortholog assignments, and as an ortholog group could encompass multiple contigs for a given species, this count was then calculated as the sum of all underlying contig read counts.

Read counts were analyzed in R v3.3.2, first filtering to remove genes with no counts or low count genes that did not have at least one count per million reads in 12 or more of the total gill libraries. The filtered gene sets were then analyzed using the Bioconductor package edgeR v3.16.5 [59] for broad trends to evaluate data integrity. This initial evaluation by MDS (multidimensional scaling) plot and hierarchical clustering analysis identified an outlier in the heat stressed *C. rastrospinosus* (Additional file 7: Figure S2), which was removed from further consideration. Differentially expressed genes were identified within species between control and heat stressed specimens using contrasts in the confines of a GLM (Generalized Linear Model) that incorporated both species and heat treatment, using a 0.01 global FDR (false discovery rate) adjusted *p* value and a log_2_FC threshold of 1. As a result, the study focused on moderate-to-high levels of differential expression and small differences in expression, more subtle changes that may lie below the 1 log2FC threshold, were not examined. The GLM followed the “each treatment combination as a group” approach described in the edgeR user guide. edgeR itself fits a negative binomial model to the dataset and estimates dispersion as the Biological Coefficient of Variability (BCV) before carrying out the analysis. The model fit was therefore first evaluated by inspection of the BCV plot and estimate of common dispersion, both of which were found to be within acceptable ranges for studies on wild-caught specimens (Additional file 2).

Following identification of differentially expressed genes, comparisons were primarily focused on *E. maclovinus* and *C. rastrospinosus* as *P. borchgrevinki* showed an extraordinarily muted response. To put the sets of differentially expressed genes found in *E. maclovinus* and *C. rastrospinosus* into biological context, we relied on Gene Ontology (GO) enrichment analysis carried out using the R package GOSeq v1.26 [60] with an FDR adjusted *p* value threshold of 0.05 and excluding any enriched GO terms associated with fewer than 20 genes. GO enrichment analysis was carried out for both the shared and species-specific sets of genes as well as for the complete set of each species’ responding genes comprising their global responses. When analyzing gene sets specific to a species we used the annotation specific to that species to test for enrichment, while we merged the annotation information determined from both species to test the shared set of common responding genes from *E. maclovinus* and *C. rastrospinosus*. The residual gill reads for each species not accounted for by the orthologous sets were mapped against the leftover segment of the respective species’ transcriptome to determine whether these showed a consistent biological response (presented in Additional file 8).

## Additional files

Additional file 1: **Table S1.** Overview of the tissues used in each species’ reference transcriptome and their respective RIN values. **Table S2.** Sequencing and Assembly Metrics. **Table S3.** Gill Sequencing and Mapping Results – Read counts. **Table S4.** Gill Sequencing and Mapping Results – Percentages. **Table S5.** *E. maclovinus* specific enrichment. **Table S6.** enrichment for the transcripts shared in the *E. maclovinus* and *C. rastrospinosus* responses. **Table S7.** *C. rastrospinosus* specific enrichment. **Table S8.** GO terms enriched from the global *E. maclovinus* transcriptional response to heat. **Table S9.** GO terms enriched from the global *C. rastrospinosus* transcriptional response to heat. (DOCX 96 kb)

Additional file 2: Corroborating the Lack of *P. borchgrevinki* Response Using limma and Voom. (DOCX 232 kb)

Additional file 3: Investigating the Role of CPM Threshold on the Detected Biological Response. (DOCX 21 kb)

Additional file 4: Investigating the Role of the FDR Threshold on the Detected Biological Response. (DOCX 19 kb)

Additional file 5: **Figure S1.** Overview of the bioinformatics workflow used in the analysis. (TIF 13925 kb)

Additional file 6: Trimmomatic Settings. (DOCX 19 kb)

Additional file 7: **Figure S2.** MDS plot and Hierarchical Clustering analysis used to identify the *C. rastrospinosus* outlier. (TIF 22467 kb)

Additional file 8: Evaluating the Biological Content of the Residual Reads. (DOCX 29 kb)

## Abbreviations

CSR: Cellular stress response
CTMax: Critical thermal maximum
ER: Endoplasmic reticulum
HSF: Heat shock factor
HSR: Heat shock response
MAPK: Mitogen-activated protein kinase
UPR: Unfolded protein response
