# Supplementary material for: Evolution in chronic cold: varied loss of cellular response to heat in Antarctic notothenioid fish
Source: BMC Evol Biol. 2018 Sep 19;18:143. doi: 10.1186/s12862-018-1254-6 (PMC6146603; doi:10.1186/s12862-018-1254-6)
Supplement: Supplementary file 1 — Table S1. Overview of the tissues used in each species’ reference transcriptome and their respective RIN values. Table S2. Sequencing and Assembly Metrics. Table S3. Gill Sequencing and Mapping Results – Read counts. Table S4. Gill Sequencing and Mapping Results – Percentages. Table S5. E. maclovinus specific enrichment. Table S6. enrichment for the transcripts shared in the E. maclovinus and C. rastrospinosus responses. Table S7. C. rastrospinosus specific enrichment. Table S8. GO terms enriched from the global E. maclovinus transcriptional response to heat. Table S9. GO terms enriched from the global C. rastrospinosus transcriptional response to heat. (DOCX 96 kb) [file 12862_2018_1254_MOESM1_ESM.docx]

**Additional file 1 Tables**

Table S1. Tissue content and quality of RNA pools used to generate the reference transcriptomes

|  | ***E. maclovinus*** | | | | | | | ***P. borchgrevinki*** | | | | | | | ***C. rastrospinosus*** | | | | | | |
| --- | --- | --- | --- | --- | --- | --- | --- | --- | --- | --- | --- | --- | --- | --- | --- | --- | --- | --- | --- | --- | --- |
|  | Control | | CTM+2hr | | CTM+4hr | | RIN | Control | | CTM+2hr | | CTM+4hr | | RIN | Control | | CTM+2hr | | CTM+4hr | | RIN |
| **Tissue** | #1 | #2 | #1 | #2 | #1 | #2 |  | ♀ | ♂ | ♀ | ♂ | ♀ | ♂ |  | ♀ | ♂ | ♀ | ♂ | ♂ | ♂ |  |
|  |  |  |  |  |  |  |  |  |  |  |  |  |  |  |  |  |  |  |  |  |  |
| **Liver** | ✓ | ✓ | ✓ | ✓ | ✓ | ✓ | 8 | ✓ | ✓ | ✓ | ✓ | ✓ | ✓ | 8.1 | ✓ | ✓ | ✓ | ✓ | ✓ | ✓ | 9 |
| **Gill** | ✓ | ✓ | ✓ | ✓ | ✓ | ✓ | 8.4 | ✓ | ✓ | ✓ | ✓ | ✓ | ✓ | 7.1 | ✓ | ✓ | ✓ | ✓ | ✓ | ✓ | 8 |
| **Brain** | ✓ | ✓ | ✓ | ✓ | ✓ | ✓ | 8.4 | ✓ | N/A | N/A | N/A | N/A | N/A | 7.5 | ✓ | ✓ | ✓ | ✓ | ✓ | ✓ | 7.7 |
| **Spleen*** | ✓ | ✓ | ✓ | ✓ | ✓ | ✓ | 6.1 | ✓ | ✓ | ✓ | ✓ | ✓ | ✓ | 7.7 | ✓ | ✓ | ✓ | ✓ | ✓ | ✓ | 7.1 |
| **Heart ^+^** | ✓ | ✓ | N/A | N/A | N/A | N/A | 7.5 | ✓ | ✓ | ✓ | ✓ | ✓ | ✓ | 8.4 | ✓ | ✓ | ✓ | ✓ | ✓ | ✓ | 8.1 |
| **Head ^+^**  **Kidney** | ✓ | ✓ | N/A | N/A | N/A | N/A | 6.6 | ✓ | ✓ | ✓ | ✓ | ✓ | ✓ | 8.3 | ✓ | ✓ | ✓ | ✓ | ✓ | ✓ | 8.7 |
| **Muscle** | ✓ | ✓ | ✓ | ✓ | ✓ | ✓ | 7 | ✓ | ✓ | ✓ | ✓ | ✓ | ✓ | 9 | ✓ | ✓ | ✓ | ✓ | ✓ | ✓ | 8.4 |
| **Stomach** | ✓ | ✓ | ✓ | ✓ | ✓ | ✓ | 7.1 | ✓ | ✓ | ✓ | ✓ | N/A | ✓ | 9.4 | ✓ | ✓ | ✓ | ✓ | ✓ | ✓ | 8.4 |
| **Intestine*** | ✓ | ✓ | ✓ | ✓ | ✓ | ✓ | 7.7 | N/A | ✓ | N/A | ✓ | ✓ | N/A | 7.1 | ✓ | ✓ | ✓ | ✓ | ✓ | ✓ | 8.9 |
| **Eye Cup** | ✓ | ✓ | ✓ | ✓ | ✓ | ✓ | 8.5 | ✓ | N/A | N/A | ✓ | N/A | ✓ | 7.4 | ✓ | ✓ | ✓ | ✓ | ✓ | ✓ | 7.5 |
| **Lens** | ✓ | ✓ | ✓ | ✓ | ✓ | ✓ | 7.9 | ✓ | ✓ | ✓ | ✓ | ✓ | ✓ | 7.6 | ✓ | ✓ | ✓ | ✓ | ✓ | ✓ | 8.7 |
|  |  |  |  |  |  |  |  |  |  |  |  |  |  |  |  |  |  |  |  |  |  |
| Merged |  |  |  |  |  |  | 7.8 |  |  |  |  |  |  | 7.7 |  |  |  |  |  |  | 8.4 |

As available, two individuals of each species were selected for each treatment in order to provide a fuller set of transcriptional responses. Where possible these were from male and female specimens in order to incorporate any gender driven differences in gene expression. Gender was not determined for the juvenile *E. maclovinus* specimens and thus two specimens were selected at random, and only male *C. rastrospinosus* specimens were collected from specimens four hours after their CTMax.

* Intestine and spleen were extracted together for *E. maclovinus* due to the small size of the spleen in juveniles

**^+^** Heart and Head Kidney were not dissected from the Juvenile specimens given their size, instead RNA was isolated from two previously collected adult samples

Table S2. Sequencing and Assembly Metrics

|  |  | ***E. maclovinus*** |  | ***P. borchgrevinki*** |  | ***C. rastrospinosus*** |
| --- | --- | --- | --- | --- | --- | --- |
|  |  |  |  |  |  |  |
| **Sequenced Reads** |  | 60,927,159 |  | 63,735,228 |  | 67,052,170 |
| **Filtered Reads** |  | 41,958,480 |  | 43,009,135 |  | 43,227,223 |
|  |  |  |  |  |  |  |
| **Preliminary Assembly** | | | | | | |
| **No. of Contigs** |  | 455,588 |  | 428,838 |  | 538,182 |
| **Mean Length** |  | 829 |  | 847 |  | 670 |
| **Median Length** |  | 431 |  | 426 |  | 371 |
| **N50** |  | 1477 |  | 1589 |  | 1002 |
|  |  |  |  |  |  |  |
| **Filtered Assembly Statistics (>1 FPKM)** | | | | | | |
| **No. of Contigs** |  | 157,790 |  | 143,700 |  | 177,320 |
| **Mean Length** |  | 814 |  | 859 |  | 709 |
| **Median Length** |  | 374 |  | 384 |  | 373 |
| **N50** |  | 1624 |  | 1754 |  | 1140 |
|  |  |  |  |  |  |  |
| **Orthologous Set** | | | | | | |
| **contigs assigned to orthologs** |  | 18,697 |  | 18,083 |  | 18,806 |
| **Mean Length** |  | 2203 |  | 2153 |  | 2014 |
| **Median Length** |  | 1924 |  | 1860 |  | 1735 |
| **N50** |  | 2802 |  | 2764 |  | 2571 |
|  |  |  |  |  |  |  |
| **Leftover Set** | | | | | | |
| **No. of Contigs** |  | 139,569 |  | 126,065 |  | 158,943 |
| **Mean Length** |  | 613 |  | 652 |  | 541 |
| **Median Length** |  | 349 |  | 356 |  | 351 |
| **N50** |  | 749 |  | 917 |  | 544 |

Table S3. Gill Sequencing and Mapping Results – Read counts

| **Specimen** |  | **Ctrl 1** | **Ctrl 2** | **Ctrl 3** | **Ctrl 4** | **CTMax 1** | **CTMax 2** | **CTMax 3** | **CTMax 4** |
| --- | --- | --- | --- | --- | --- | --- | --- | --- | --- |
|  |  |  |  |  |  |  |  |  |  |
| ***E. maclovinus*** | |  |  |  |  |  |  |  |  |
| Sequenced | | 37,903,356 | 31,810,728 | 31,754,832 | 33,211,741 | 32,320,532 | 32,567,627 | 36,041,294 | 33,237,472 |
| Filtered | | 34,092,294 | 28,509,863 | 28,442,421 | 29,793,637 | 29,046,081 | 29,216,348 | 32,282,910 | 29,795,155 |
| Orthologous | | 20,087,622 | 16,431,125 | 15,782,658 | 17,322,170 | 16,438,774 | 16,980,836 | 17,996,707 | 17,285,390 |
| Leftover | | 10,749,088 | 9,390,661 | 9,541,593 | 9,478,695 | 9,981,150 | 9,587,601 | 11,555,269 | 9,584,268 |
| None | | 3,255,584 | 2,688,077 | 3,118,170 | 2,992,772 | 2,626,157 | 2,647,911 | 2,730,934 | 2,925,497 |
|  |  |  |  |  |  |  |  |  |  |
| ***P. borchgrevinki*** | |  |  |  |  |  |  |  |  |
| Sequenced | | 32,726,434 | 36,331,266 | 32,478,731 | 32,691,619 | 32,146,375 | 32,442,365 | 35,770,955 | 39,064,485 |
| Filtered | | 29,346,268 | 32,709,128 | 29,118,606 | 29,261,354 | 28,753,102 | 29,027,943 | 32,048,354 | 34,766,445 |
| Orthologous | | 15,283,338 | 6,293,960 | 15,679,437 | 16,013,658 | 14,646,661 | 15,509,687 | 16,562,788 | 18,575,139 |
| Leftover | | 9,807,314 | 4,051,520 | 10,108,575 | 9,566,120 | 9,620,291 | 9,811,326 | 10,997,425 | 11,704,857 |
| None | | 4,255,616 | 22,363,648 | 3,330,594 | 3,681,576 | 4,486,150 | 3,706,930 | 4,488,141 | 4,486,449 |
|  |  |  |  |  |  |  |  |  |  |
| ***C. rastrospinosus*** | |  |  |  |  |  |  |  |  |
| Sequenced | | 34,275,786 | 30,775,541 | 33,756,569 | 33,268,267 | 30,356,437 | 30,361,682 | 36,217,260 | 31,745,237 |
| Filtered | | 30,481,982 | 27,559,969 | 30,175,579 | 29,704,819 | 27,132,002 | 27,194,390 | 32,337,935 | 28,344,438 |
| Orthologous | | 15,852,834 | 14,373,389 | 15,533,332 | 15,261,602 | 14,910,130 | 14,493,901 | 17,263,401 | 14,974,071 |
| Leftover | | 9,837,887 | 8,948,738 | 9,637,215 | 9,513,862 | 8,399,625 | 8,686,618 | 10,394,085 | 8,882,036 |
| None | | 4,791,261 | 4,237,842 | 5,005,032 | 4,929,355 | 3,822,247 | 4,013,871 | 4,680,449 | 4,488,331 |

Sequenced – number of raw reads

Filtered – number of quality filtered reads

Orthologous – number of quality filtered reads mapping to the orthologous gene set

Leftover – number of quality filtered reads mapping to the leftover gene set

None – number of quality filtered read not mapping to the orthologous or leftover set

*P. borchgrevinki* control 2 with read counts in red numbers was identified as anomalous given the low mapping efficiency, and thus was excluded from further analysis.

*C. rastrospinosus* CTMax4 with read counts in blue numbers was subsequently identified as an outlier in MDS plot and PCA analysis (S Fig. 2), and thus was excluded from downstream analysis.

Table S4. Gill Sequencing and Mapping Results - Percentages

| **Specimen** |  | **Ctrl 1** | **Ctrl 2** | **Ctrl 3** | **Ctrl 4** | **CTMax 1** | **CTMax 2** | **CTMax 3** | **CTMax 4** | **Average** |
| --- | --- | --- | --- | --- | --- | --- | --- | --- | --- | --- |
|  |  |  |  |  |  |  |  |  |  |  |
| ***E. maclovinus*** | |  |  |  |  |  |  |  |  |  |
| Orthologous | | 58.9% | 57.6% | 55.4% | 58.1% | 56.5% | 58.1% | 55.7% | 58.0% | **57.3%** |
| Leftover | | 31.5% | 32.9% | 33.5% | 31.8% | 34.3% | 32.8% | 35.7% | 32.1% | **33.1%** |
| None | | 9.5% | 9.4% | 10.9% | 10.0% | 9.0% | 9.0% | 8.4% | 9.8% | **9.5%** |
|  |  |  |  |  |  |  |  |  |  |  |
| ***P. borchgrevinki*** | |  |  |  |  |  |  |  |  |  |
| Orthologous | | 52.0% | 19.2% | 53.8% | 54.7% | 50.9% | 53.4% | 51.6% | 52.3% | **52.8%** |
| Leftover | | 33.4% | 12.3% | 34.7% | 32.6% | 33.4% | 33.7% | 34.3% | 33.6% | **33.7%** |
| None | | 14.5% | 68.3% | 11.4% | 12.5% | 15.6% | 12.7% | 14.0% | 12.9% | **13.4%** |
|  |  |  |  |  |  |  |  |  |  |  |
| ***C. rastrospinosus*** | |  |  |  |  |  |  |  |  |  |
| Orthologous | | 52.0% | 52.1% | 51.4% | 51.3% | 54.9% | 53.2% | 53.3% | 52.8% | **52.6%** |
| Leftover | | 32.2% | 32.4% | 31.9% | 32.0% | 30.9% | 31.9% | 32.1% | 31.3% | **31.8%** |
| None | | 15.7% | 15.3% | 16.5% | 16.5% | 14.0% | 14.7% | 14.4% | 15.8% | **15.4%** |

Orthologous – percent of quality filtered reads mapping to the orthologous gene set

Leftover – percent of quality filtered reads mapping to the leftover gene set

None – percent of quality filtered read not mapping to the orthologous or leftover set

*P. borchgrevinki* control 2 with read mapping percentages in red numbers was identified as anomalous given the low mapping efficiency, and thus was excluded from further analysis.

*C. rastrospinosus* CTMax4 with read counts in blue numbers was subsequently identified as an outlier in MDS plot and PCA analysis (S Fig. 2), and thus was excluded from downstream analysis

Table S5. *E. maclovinus* specific enriched GO terms

| **GO ID** | **Description** | **P value** | **FDR** |  | **up** | **down** |
| --- | --- | --- | --- | --- | --- | --- |
|  |  |  |  |  |  |  |
| **Biological Process** | | | | | | |
| GO:0006457 | protein folding | 5.67e-10 | 3.09e-06 |  | 36 | 1 |
| GO:0046165 | alcohol biosynthetic process | 3.31e-08 | 1.35e-04 |  | 18 | 4 |
| GO:1901617 | organic hydroxy compound biosynthetic process | 1.42e-05 | 2.59e-02 |  | 18 | 4 |
|  |  |  |  |  |  |  |
| **Cellular Component** | | | | | | |
| GO:0044432 | endoplasmic reticulum part | 3.73e-11 | 6.10e-07 |  | 88 | 35 |
| GO:0005789 | endoplasmic reticulum membrane | 5.41e-10 | 3.09e-06 |  | 65 | 31 |

Table S6. Enriched GO terms for the transcripts shared in the *E. maclovinus* and *C. rastrospinosus* responses

| **GO ID** | **Description** | ***P value*** | **FDR** |  | ***E. maclovinus*** | |  | ***C. rastrospinosus*** | |
| --- | --- | --- | --- | --- | --- | --- | --- | --- | --- |
|  |  |  |  |  | **up** | **down** |  | **up** | **Down** |
|  |  |  |  |  |  |  |  |  |  |
| **Biological Process** | | | | | | | | | |
| GO:0065007 | biological regulation | 8.10e-07 | 0.0032 |  | 130 | 87 |  | 127 | 90 |
| GO:0050794 | regulation of cellular process | 9.39e-07 | 0.0032 |  | 121 | 80 |  | 119 | 82 |
| GO:0010556 | regulation of macromolecule biosynthetic process | 1.43e-06 | 0.0040 |  | 60 | 40 |  | 55 | 45 |
| GO:0060255 | regulation of macromolecule metabolic process | 3.63e-06 | 0.0075 |  | 77 | 53 |  | 74 | 56 |
| GO:0031326 | regulation of cellular biosynthetic process | 3.76e-06 | 0.0075 |  | 61 | 40 |  | 55 | 46 |
| GO:0019222 | regulation of metabolic process | 4.29e-06 | 0.0075 |  | 82 | 61 |  | 79 | 64 |
| GO:0010468 | regulation of gene expression | 4.43e-06 | 0.0075 |  | 63 | 42 |  | 59 | 46 |
| GO:0009889 | regulation of biosynthetic process | 6.28e-06 | 0.0087 |  | 61 | 40 |  | 55 | 46 |
| GO:0007165 | signal transduction | 6.58e-06 | 0.0087 |  | 59 | 38 |  | 61 | 36 |
| GO:0035556 | intracellular signal transduction | 7.37e-06 | 0.0087 |  | 40 | 12 |  | 39 | 13 |
| GO:0080090 | regulation of primary metabolic process | 7.63e-06 | 0.0087 |  | 74 | 53 |  | 70 | 57 |
| GO:0031323 | regulation of cellular metabolic process | 7.97e-06 | 0.0087 |  | 74 | 57 |  | 70 | 61 |
| GO:2000112 | regulation of cellular macromolecule biosynthetic process | 8.22e-06 | 0.0087 |  | 57 | 39 |  | 51 | 45 |
| GO:2001141 | regulation of RNA biosynthetic process | 2.93e-05 | 0.027 |  | 51 | 34 |  | 45 | 40 |
| GO:0050789 | regulation of biological process | 3.33e-05 | 0.029 |  | 123 | 81 |  | 121 | 83 |
| GO:0006355 | regulation of transcription, DNA-templated | 4.93e-05 | 0.040 |  | 50 | 34 |  | 44 | 40 |
| GO:1903506 | regulation of nucleic acid-templated transcription | 4.93e-05 | 0.040 |  | 50 | 34 |  | 44 | 40 |
| GO:0050896 | response to stimulus | 6.01e-05 | 0.044 |  | 63 | 39 |  | 64 | 38 |
|  |  |  |  |  |  |  |  |  |  |
| **Molecular Function** | | | | | | | | | |
| GO:0001071 | nucleic acid binding transcription factor activity | 2.15e-07 | 0.0018 |  | 26 | 13 |  | 23 | 16 |
| GO:0003700 | transcription factor activity, sequence-specific DNA binding | 2.15e-07 | 0.0018 |  | 26 | 13 |  | 23 | 16 |
| GO:0043565 | sequence-specific DNA binding | 9.15e-07 | 0.0032 |  | 21 | 15 |  | 19 | 17 |
| GO:0000981 | RNA polymerase II transcription factor activity, sequence-specific DNA binding | 1.96e-05 | 0.019 |  | 14 | 9 |  | 14 | 9 |

Table S7. *C. rastrospinosus* specific enriched GO terms

| **GO ID** | **Description** | **P value** | **FDR** |  | **up** | **down** |
| --- | --- | --- | --- | --- | --- | --- |
|  |  |  |  |  |  |  |
| **Biological Process** | | | | | | |
| GO:0044707 | single-multicellular organism process | 9.70e-10 | 1.08e-05 |  | 74 | 81 |
| GO:0032501 | multicellular organismal process | 1.32e-09 | 1.08e-05 |  | 75 | 81 |
| GO:0030335 | positive regulation of cell migration | 2.66e-07 | 1.38e-03 |  | 31 | 9 |
| GO:2000147 | positive regulation of cell motility | 3.37e-07 | 1.38e-03 |  | 31 | 9 |
| GO:0051272 | positive regulation of cellular component movement | 4.92e-07 | 1.61e-03 |  | 31 | 9 |
| GO:0040017 | positive regulation of locomotion | 6.91e-07 | 1.89e-03 |  | 31 | 9 |
| GO:0030334 | regulation of cell migration | 3.28e-06 | 7.71e-03 |  | 39 | 15 |
| GO:0048585 | negative regulation of response to stimulus | 5.57e-06 | 1.01e-02 |  | 46 | 38 |
| GO:0040012 | regulation of locomotion | 9.01e-06 | 1.34e-02 |  | 40 | 18 |
| GO:2000145 | regulation of cell motility | 1.21e-05 | 1.66e-02 |  | 39 | 15 |
| GO:0051239 | regulation of multicellular organismal process | 1.51e-05 | 1.78e-02 |  | 75 | 61 |
| GO:0009966 | regulation of signal transduction | 2.13e-05 | 2.06e-02 |  | 84 | 55 |
| GO:0042127 | regulation of cell proliferation | 2.31e-05 | 2.11e-02 |  | 51 | 31 |
| GO:0030154 | cell differentiation | 3.34e-05 | 2.73e-02 |  | 58 | 44 |
| GO:0051270 | regulation of cellular component movement | 3.49e-05 | 2.73e-02 |  | 39 | 15 |
| GO:0006954 | inflammatory response | 4.15e-05 | 2.96e-02 |  | 19 | 12 |
| GO:0009968 | negative regulation of signal transduction | 4.58e-05 | 3.13e-02 |  | 38 | 29 |
|  |  |  |  |  |  |  |
| **Molecular Function** | | | | | | |
| GO:0005102 | receptor binding | 8.97e-06 | 1.34e-02 |  | 46 | 21 |
| GO:0004930 | G-protein coupled receptor activity | 2.99e-05 | 2.59e-02 |  | 15 | 9 |
|  |  |  |  |  |  |  |
| **Cellular Component** | | | | | | |
| GO:0005576 | extracellular region | 4.77e-06 | 9.80e-03 |  | 37 | 26 |
| GO:0005615 | extracellular space | 1.63e-05 | 1.79e-02 |  | 38 | 22 |
| GO:0005887 | integral component of plasma membrane | 4.15e-05 | 2.96e-02 |  | 36 | 23 |
| GO:0031226 | intrinsic component of plasma membrane | 6.01e-05 | 3.94e-02 |  | 37 | 24 |

Table S8. GO terms enriched from the *E. maclovinus* transcriptional response to heat

| **GO ID** | **Description** | **P value** | **FDR** |  | **up** | **down** |
| --- | --- | --- | --- | --- | --- | --- |
|  |  |  |  |  |  |  |
| **Biological Process** | | | | | | |
| GO:0006457 | protein folding | 1.90e-08 | 1.03e-04 |  | 39 | 1 |
| GO:0046165 | alcohol biosynthetic process | 9.93e-07 | 2.70e-03 |  | 19 | 4 |
| GO:0006694 | steroid biosynthetic process | 1.33e-05 | 1.99e-02 |  | 17 | 4 |
|  |  |  |  |  |  |  |
| **Molecular Function** | | | | | | |
| GO:0043565 | sequence-specific DNA binding | 5.82e-10 | 9.52e-06 |  | 43 | 63 |
| GO:0000976 | transcription regulatory region sequence-specific DNA binding | 5.64e-07 | 1.84e-03 |  | 28 | 36 |
| GO:0000975 | regulatory region DNA binding | 8.89e-06 | 1.61e-02 |  | 35 | 43 |
| GO:0001067 | regulatory region nucleic acid binding | 1.02e-05 | 1.67e-02 |  | 35 | 43 |
| GO:0044212 | transcription regulatory region DNA binding | 1.66e-05 | 2.18e-02 |  | 34 | 42 |
| GO:0001071 | nucleic acid binding transcription factor activity | 1.87e-05 | 2.18e-02 |  | 51 | 46 |
| GO:0003700 | Transcription factor activity, sequence-specific DNA binding | 1.87e-05 | 2.18e-02 |  | 51 | 46 |
| GO:0000978 | RNA polymerase II core promoter proximal region sequence-specific DNA binding | 3.65e-05 | 3.98e-02 |  | 15 | 17 |
|  |  |  |  |  |  |  |
| **Cellular Component** | | | | | | |
| GO:0005789 | endoplasmic reticulum membrane | 1.72e-08 | 1.03e-04 |  | 73 | 40 |
| GO:0044432 | endoplasmic reticulum part | 2.94e-08 | 1.20e-04 |  | 97 | 44 |

Table S9. GO terms enriched from the *C. rastrospinosus* transcriptional response to heat

| **GO ID** | **Description** | **P value** | **FDR** |  | **up** | **down** |
| --- | --- | --- | --- | --- | --- | --- |
|  |  |  |  |  |  |  |
| **Biological Process** | | | | | | |
| GO:0044707 | single-multicellular organism process | 2.32E-10 | 1.99E-06 |  | 103 | 100 |
| GO:0032501 | multicellular organismal process | 2.42E-10 | 1.99E-06 |  | 104 | 101 |
| GO:0048585 | negative regulation of response to stimulus | 4.66E-09 | 1.91E-05 |  | 64 | 55 |
| GO:0006954 | inflammatory response | 1.33E-08 | 3.76E-05 |  | 31 | 16 |
| GO:0051239 | regulation of multicellular organismal process | 1.37E-08 | 3.76E-05 |  | 109 | 83 |
| GO:0030335 | positive regulation of cell migration | 3.40E-08 | 6.99E-05 |  | 40 | 11 |
| GO:2000147 | positive regulation of cell motility | 4.54E-08 | 8.24E-05 |  | 40 | 11 |
| GO:0048583 | regulation of response to stimulus | 5.85E-08 | 8.24E-05 |  | 144 | 102 |
| GO:0009966 | regulation of signal transduction | 6.02E-08 | 8.24E-05 |  | 118 | 76 |
| GO:0051272 | positive regulation of cellular component movement | 6.94E-08 | 8.76E-05 |  | 40 | 11 |
| GO:0032502 | developmental process | 7.61E-08 | 8.92E-05 |  | 177 | 166 |
| GO:0040017 | positive regulation of locomotion | 1.05E-07 | 0.00011 |  | 40 | 11 |
| GO:0033993 | response to lipid | 1.15E-07 | 0.00011 |  | 45 | 25 |
| GO:0009968 | negative regulation of signal transduction | 1.20E-07 | 0.00011 |  | 55 | 40 |
| GO:0042127 | regulation of cell proliferation | 1.24E-07 | 0.00011 |  | 73 | 41 |
| GO:0050896 | response to stimulus | 1.47E-07 | 0.00012 |  | 178 | 122 |
| GO:0044767 | single-organism developmental process | 4.14E-07 | 0.00029 |  | 162 | 157 |
| GO:0001071 | nucleic acid binding transcription factor activity | 4.51E-07 | 0.00029 |  | 44 | 45 |
| GO:0006952 | defense response | 4.64E-07 | 0.00029 |  | 55 | 26 |
| GO:0023051 | regulation of signaling | 5.27E-07 | 0.00032 |  | 124 | 79 |
| GO:0007166 | cell surface receptor signaling pathway | 1.05E-06 | 0.00059 |  | 96 | 65 |
| GO:0023057 | negative regulation of signaling | 1.46E-06 | 0.00079 |  | 55 | 41 |
| GO:0030154 | cell differentiation | 1.55E-06 | 0.00080 |  | 77 | 62 |
| GO:0040012 | regulation of locomotion | 1.56E-06 | 0.00080 |  | 54 | 22 |
| GO:2000145 | regulation of cell motility | 2.12E-06 | 0.0010 |  | 53 | 18 |
| GO:0010648 | negative regulation of cell communication | 2.20E-06 | 0.0010 |  | 56 | 41 |
| GO:0010646 | regulation of cell communication | 3.09E-06 | 0.0014 |  | 123 | 80 |
| GO:0030334 | regulation of cell migration | 3.48E-06 | 0.0015 |  | 51 | 17 |
| GO:0051270 | regulation of cellular component movement | 4.42E-06 | 0.0018 |  | 54 | 18 |
| GO:1901700 | response to oxygen-containing compound | 5.65E-06 | 0.0022 |  | 58 | 39 |
| GO:0051241 | negative regulation of multicellular organismal process | 7.17E-06 | 0.0028 |  | 47 | 35 |
| GO:0015849 | organic acid transport | 1.45E-05 | 0.0051 |  | 21 | 8 |
| GO:0046942 | carboxylic acid transport | 1.45E-05 | 0.0051 |  | 21 | 8 |
| GO:0015711 | organic anion transport | 1.47E-05 | 0.0051 |  | 26 | 11 |
| GO:0009653 | anatomical structure morphogenesis | 2.57E-05 | 0.0081 |  | 59 | 55 |
| GO:0071396 | cellular response to lipid | 3.56E-05 | 0.010 |  | 22 | 13 |
| GO:0006950 | response to stress | 3.59E-05 | 0.010 |  | 115 | 78 |
| GO:0048513 | animal organ development | 3.60E-05 | 0.010 |  | 53 | 48 |
| GO:0048869 | cellular developmental process | 3.79E-05 | 0.010 |  | 101 | 87 |
| GO:0051174 | regulation of phosphorus metabolic process | 4.11E-05 | 0.011 |  | 73 | 43 |
| GO:0071407 | cellular response to organic cyclic compound | 4.34E-05 | 0.012 |  | 21 | 12 |
| GO:0004930 | G-protein coupled receptor activity | 4.71E-05 | 0.012 |  | 19 | 10 |
| GO:0014070 | response to organic cyclic compound | 5.87E-05 | 0.014 |  | 39 | 21 |
| GO:0051240 | positive regulation of multicellular organismal process | 6.67E-05 | 0.015651 |  | 58 | 42 |
| GO:0006955 | immune response | 7.44E-05 | 0.016 |  | 39 | 28 |
| GO:0048856 | anatomical structure development | 8.34E-05 | 0.018 |  | 108 | 84 |
| GO:0042330 | taxis | 8.70E-05 | 0.018 |  | 22 | 6 |
| GO:0019220 | regulation of phosphate metabolic process | 9.76E-05 | 0.020 |  | 72 | 41 |
| GO:0042221 | response to chemical | 0.00010 | 0.021 |  | 95 | 61 |
| GO:0007186 | G-protein coupled receptor signaling pathway | 0.00011 | 0.022 |  | 32 | 10 |
| GO:0043408 | regulation of MAPK cascade | 0.00012 | 0.023 |  | 34 | 20 |
| GO:0008285 | negative regulation of cell proliferation | 0.00013 | 0.025 |  | 28 | 25 |
| GO:0004872 | chemotaxis | 0.00014 | 0.027 |  | 45 | 28 |
| GO:0070887 | cellular response to chemical stimulus | 0.00015 | 0.028 |  | 55 | 40 |
| GO:0050678 | regulation of epithelial cell proliferation | 0.00016 | 0.029 |  | 19 | 10 |
| GO:0048598 | embryonic morphogenesis | 0.00017 | 0.031 |  | 19 | 21 |
| GO:2000026 | regulation of multicellular organismal development | 0.00020 | 0.034 |  | 68 | 50 |
| GO:0010033 | response to organic substance | 0.00020 | 0.034 |  | 79 | 49 |
| GO:0006820 | anion transport | 0.00020 | 0.034 |  | 29 | 12 |
| GO:0065007 | biological regulation | 0.00021 | 0.034 |  | 350 | 318 |
| GO:0048545 | response to steroid hormone | 0.00023 | 0.036 |  | 25 | 9 |
| GO:0034097 | response to cytokine | 0.00030 | 0.045 |  | 25 | 12 |
| GO:0009725 | response to hormone | 0.00031 | 0.045 |  | 35 | 18 |
| GO:0002237 | response to molecule of bacterial origin | 0.00031 | 0.045 |  | 23 | 6 |
| GO:0042325 | regulation of phosphorylation | 0.00035 | 0.049 |  | 63 | 36 |
|  |  |  |  |  |  |  |
| **Molecular Function** | | | | | | |
| GO:0005102 | receptor binding | 3.10E-07 | 0.00023 |  | 58 | 32 |
| GO:0003700 | transcription factor activity, sequence-specific DNA binding | 4.51E-07 | 0.00029 |  | 44 | 45 |
| GO:0043565 | sequence-specific DNA binding | 4.56E-06 | 0.0018 |  | 34 | 48 |
| GO:0005126 | cytokine receptor binding | 1.96E-05 | 0.0064 |  | 11 | 11 |
| GO:0015291 | secondary active transmembrane transporter activity | 5.16E-05 | 0.013 |  | 20 | 7 |
| GO:0004888 | transmembrane signaling receptor activity | 6.38E-05 | 0.015 |  | 32 | 22 |
| GO:0003677 | DNA binding | 9.60E-05 | 0.020 |  | 61 | 108 |
| GO:0038023 | signaling receptor activity | 0.000142 | 0.027 |  | 36 | 24 |
| GO:0004872 | receptor activity | 0.000166 | 0.029 |  | 22 | 5 |
| GO:0000981 | RNA polymerase II transcription factor activity, sequence-specific DNA binding | 0.00017 | 0.030 |  | 22 | 26 |
|  |  |  |  |  |  |  |
| **Cellular Component** | | | | | | |
| GO:0005576 | extracellular region | 5.18E-10 | 2.83E-06 |  | 57 | 35 |
| GO:0005887 | integral component of plasma membrane | 5.23E-08 | 8.24E-05 |  | 56 | 29 |
| GO:0031226 | intrinsic component of plasma membrane | 1.56E-07 | 0.00012 |  | 57 | 30 |
| GO:0005615 | extracellular space | 2.46E-07 | 0.00019 |  | 54 | 28 |
| GO:0009986 | cell surface | 3.65E-05 | 0.010 |  | 30 | 17 |
| GO:0044459 | plasma membrane part | 5.29E-05 | 0.014 |  | 102 | 55 |
