# Supplementary material for: Evolution in chronic cold: varied loss of cellular response to heat in Antarctic notothenioid fish
Source: BMC Evol Biol. 2018 Sep 19;18:143. doi: 10.1186/s12862-018-1254-6 (PMC6146603; doi:10.1186/s12862-018-1254-6)
Supplement: Supplementary file 2 — Corroborating the Lack of P. borchgrevinki Response Using limma and Voom. (DOCX 232 kb) [file 12862_2018_1254_MOESM2_ESM.docx]

**Additional file 2,**

**Corroborating the Lack of *P. borchgrevinki* Response Using limma and Voom**

To validate our model and analysis of the three species, and the lack of response in *P. borchgrevinki* in particular, we inspected the BCV plot as well as downstream P-value histograms for the three species for signs of problems in our model.

**Figure 1, BCV Plot**


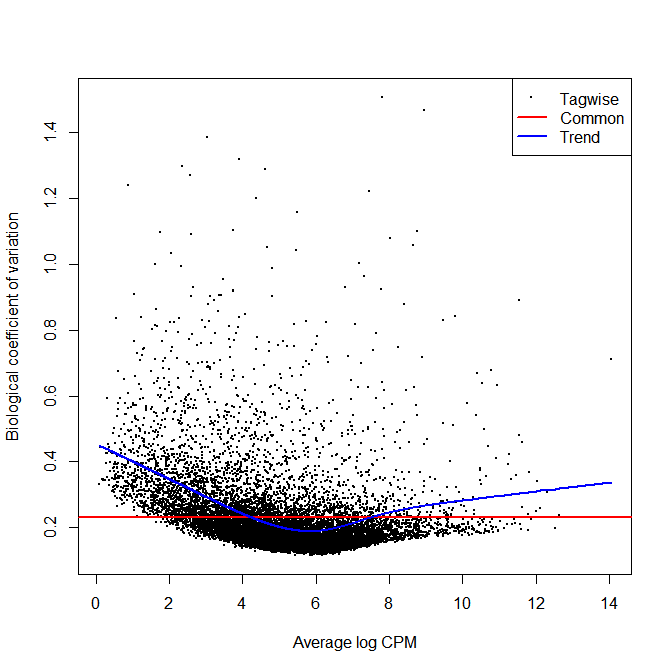


The common estimate of dispersion was 0.05335 and the BCV 0.231 both of which are well within expected levels for investigations on wild-caught specimens. Model errors will typically turn up as an inflation in the BCV or aberrant patterns on the BCV plot neither of which are apparent above in Figure 1. Looking next at the P-Value histograms from each species’ contrasts:


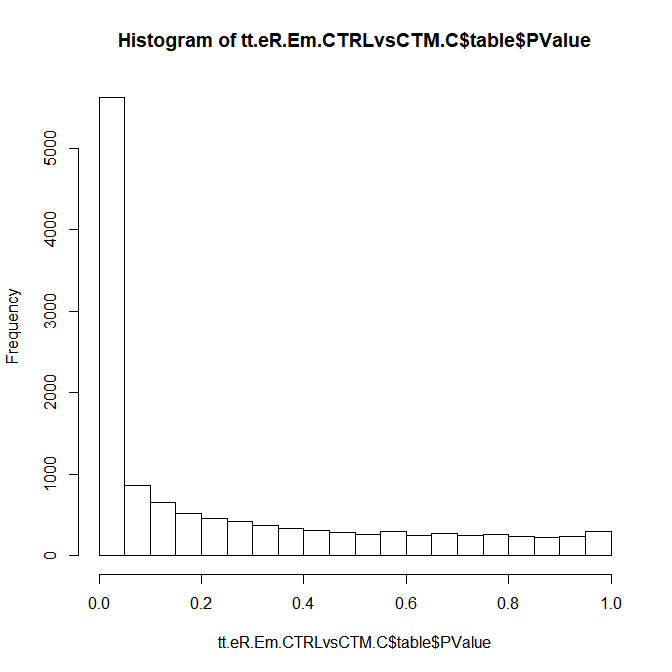


**P Value**

**Figure 2A, *E. maclovinus* CTMax vs Native P-value Histogram**


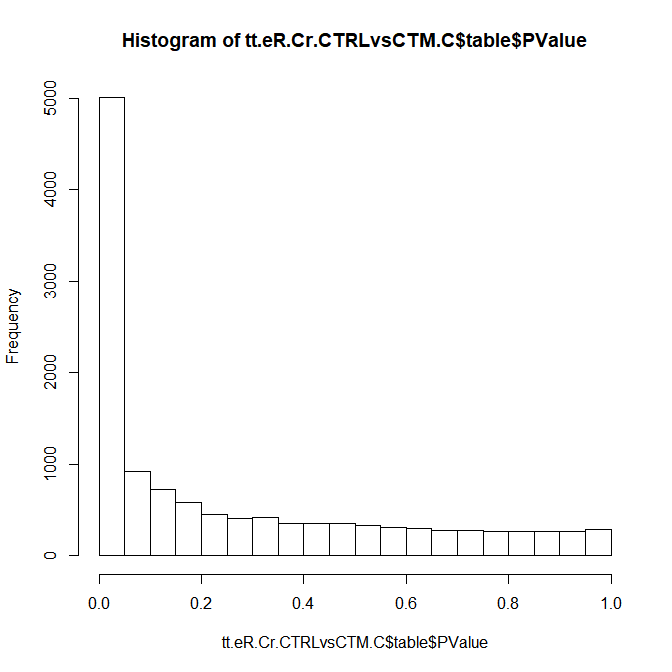


**Figure 2B, *C. rastrospinosus* CTMax vs Native P-value Histogram**

**P Value**


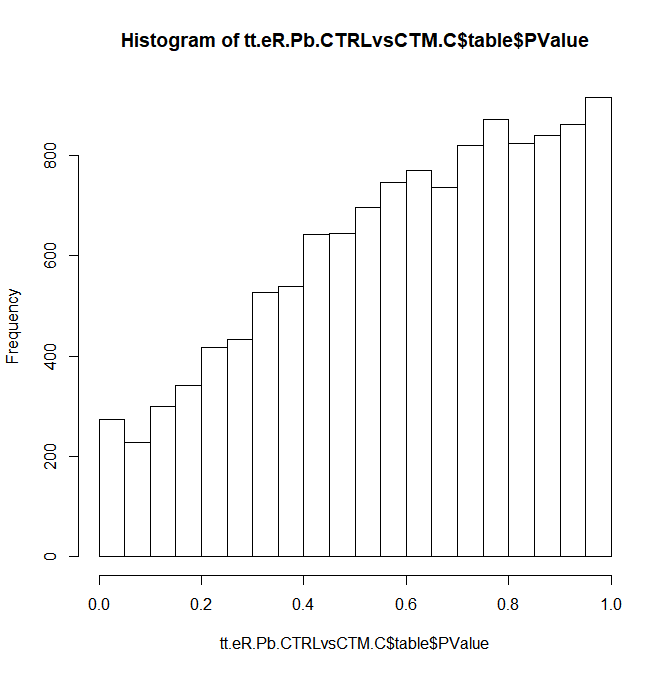


**Figure 2C, *P. borchgrevinki* CTMax vs Native P-value Histogram**

**P Value**

These show appropriate anti-conservative P-value histograms for *E. maclovinus* (Fig 2A) and *C. rastrospinosus* (Fig 2B) where strong treatment effects are present. However, the histogram for *P. borchgrevinki* does show a rising trend that could suggest a model failure. It is worth noting that for this last species, the aberrant P-value histogram coexists with a BCV plot (Figure 1) that does not suggest model problems and an MDS plot (Figure 3) that shows a lack of between group variation but otherwise does not suggest any unusual batch effects or unusual between specimen variation:

**Figure 3, *P. borchgrevinki* MDS Plot**


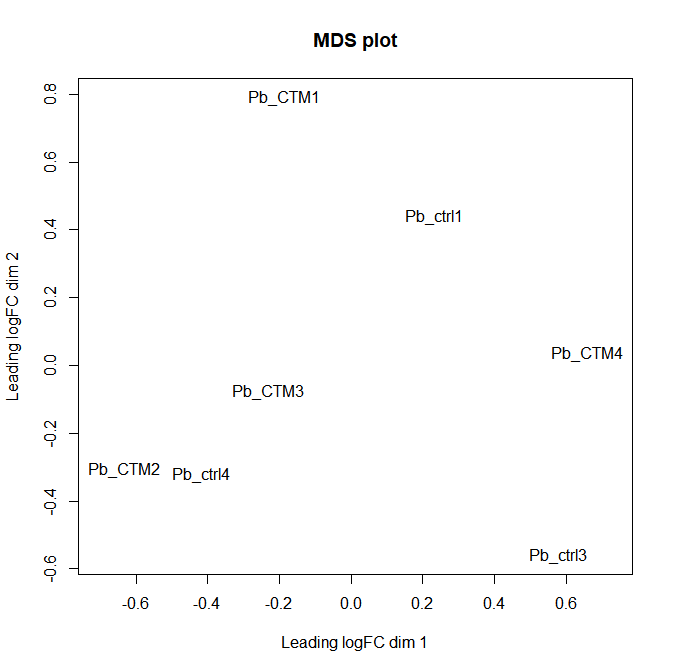


Though batch effects were not visible in this MDS plot we tested for the impact of any that may be present using RUV Seq [60] both with the empirical control gene approach and with residuals. Neither produced an improved distribution of samples based on PCA analysis nor did we find a meaningful increase in differentially expressed genes following correction for putative batch effects suggesting that none existed.

While it is possible that the trend seen in the *P. borchgrevinki* P-value histogram is due to problems with the model such as unexpected batch effects or an incorrect estimate of variance, an alternative interpretation presented by searching public knowledgebases was that P- value estimation in edgeR may be particularly sensitive to between group heteroscedasticity in the absence of a treatment effect. Limma with Voom normalization was presented as a more robust alternative under these conditions, which we used to reanalyze the dataset and verify the validity of our original findings, including the lack of response in *P. borchgrevinki*.

The analysis was carried out as presented in the main study. No and low count genes were excluded using a CPM>1 threshold of 12 libraries. We used the recommended “Analyzing as for a single factor” model design suggested in the edgeR and limma user guides though also explored a classic fully factorial design, and found no change in results. The RNA Seq reads were preprocessed with Voom run with the option to incorporate sample-specific quality weights.


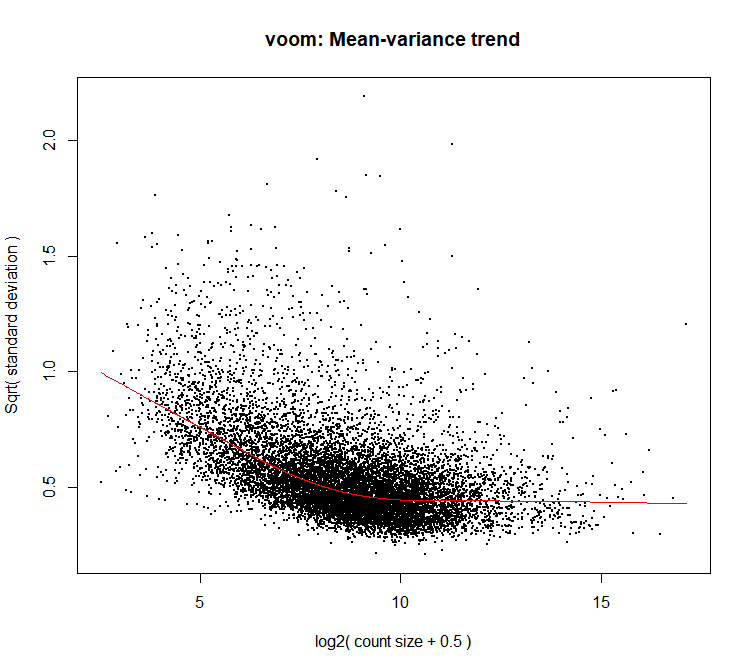


**Figure 4, Voom Mean-Variance Trend**

As before, contrasts were used to identify species specific responses in to heat stress. Due to observed differences in sensitivity between edgeR and limma we utilized 1 log_2_FC and FDR adjusted P-value < 0.05 thresholds for identifying differentially expressed genes in limma.

| **SPECIES** | **Limma DE GENES** | **edgeR DE GENES** |
| --- | --- | --- |
| ***E. maclovinus*** | 1,422 | 1,607 |
| ***C. rastrospinosus*** | 1,341 | 1,410 |
| ***P. borchgrevinki*** | 7 | 25 |

The limma estimates are more conservative than the edgeR estimates but follows the same species trends in differential expression and corroborated the lack of response in *P. borchgrevinki*. Investigation of the resulting P-value histograms shown below again shows anti-conservative histograms for *E. maclovinus* (Figure 5A) and *C. rastrospinosus* (Figure 5B) but now a more appropriate uniform distribution for *P. borchgrevinki* (Figure 5C).


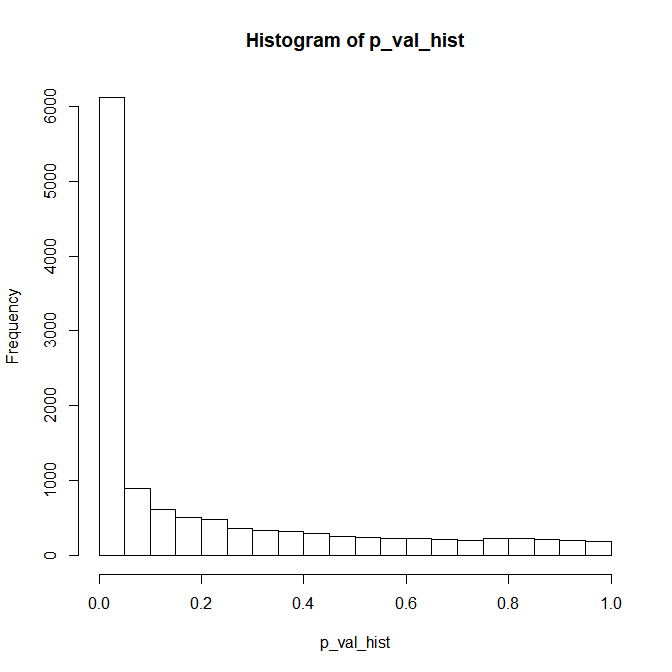


**P Value**

**Figure 5A, *E. maclovinus* CTMax vs Native P-value Histogram**


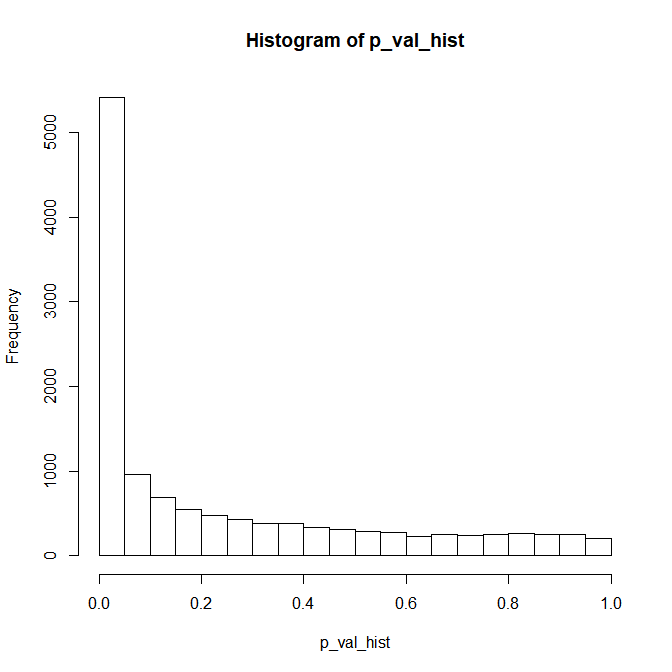


**P Value**

**Figure 5B, *C. rastrospinosus* CTMax vs Native P-value Histogram**


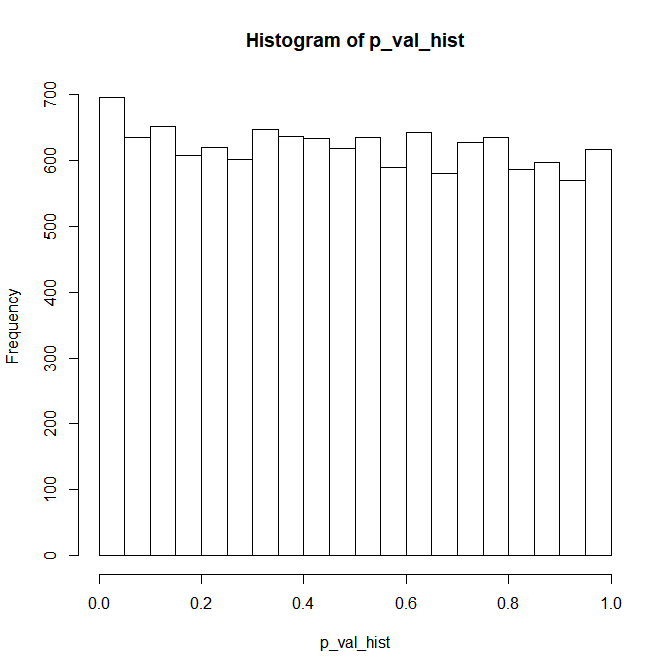


**P Value**

**Figure 5C, *P. borchgrevinki* CTMax vs Native P-value Histogram**

This separate limma analysis serves to validate the lack of response originally seen in the edgeR analysis of the *P. borchgrevinki* response. While limma detected fewer DE genes than the original edgeR analysis, this pattern is observed in all three species and likely reflects an innate biases of the differing approaches.

Finally, to validate the biological accuracy of this analysis we carried out GO enrichment analysis using GOSeq on the *E. maclovinus* species specific, *C. rastrospinosus* species specific, as well as the shared *E. maclovinus* and *C. rastrospinosus* responses which were the focus of our original analysis. Common terms are shown in bold, unique terms are greyed.

***E. maclovinus* Specific Response**

| **Limma + Voom** | |  | **edgeR** | |
| --- | --- | --- | --- | --- |
| **GO Term** | **DE genes** |  | **GO Term** | **DE Genes** |
| **Endoplasmic reticulum part** | **109** |  | **Endoplasmic Reticulum Part** | **123** |
| **Endoplasmic reticulum membrane** | **83** |  | **Endoplasmic Reticulum Membrane** | **96** |
| **Protein folding** | **35** |  | **Protein Folding** | **37** |
| **Alcohol biosynthetic process** | **22** |  | **Alcohol Biosynthetic Process** | **22** |
| **Organic hydroxy compound biosynthetic process** | **22** |  | **organic hydroxy compound biosynthetic process** | **22** |
| Small molecule biosynthetic process | 42 |  |  |  |
| Endoplasmic reticulum lumen | 21 |  |  |  |

Here, limma with Voom has captured all of the enriched terms from the original analysis with some signs of greater specificity given the addition of two further enriched terms.

***C. rastrospinosus* Specific Response**

| **Limma + Voom** | |  | **edgeR** | |
| --- | --- | --- | --- | --- |
| **GO Term** | **DE genes** |  | **GO Term** | **DE Genes** |
| **Extracellular region** | **76** |  | **Extracellular region** | **63** |
| **Extracellular space** | **74** |  | **Extracellular space** | **60** |
| **Multicellular organismal process** | **162** |  | **Multicellular organismal process** | **156** |
| **Regulation of cell proliferation** | **94** |  | **Regulation of cell proliferation** | **82** |
| **Regulation of multicellular organismal process** | **152** |  | **Regulation of multicellular organismal process** | **136** |
| **Intrinsic component of plasma membrane** | **72** |  | **Intrinsic component of plasma membrane** | **61** |
| **Integral component of plasma membrane** | **69** |  | **Integral component of plasma membrane** | **59** |
| **Positive regulation of cell migration** | **41** |  | **Positive regulation of cell migration** | **40** |
| **Positive regulation of cell motility** | **41** |  | **Positive regulation of cell motility** | **40** |
| **G-protein coupled receptor activity** | **28** |  | **G-protein coupled receptor activity** | **24** |
| **Positive regulation of cellular component movement** | **41** |  | **Positive regulation of cellular component movement** | **40** |
| **Positive regulation of locomotion** | **41** |  | **Positive regulation of locomotion** | **40** |
| **Regulation of cell migration** | **56** |  | **Regulation of cell migration** | **54** |
| **Regulation of locomotion** | **61** |  | **Regulation of locomotion** | **58** |
| **Regulation of cell motility** | **57** |  | **Regulation of cell motility** | **54** |
| **Cell differentiation** | **109** |  | **Cell differentiation** | **102** |
| **Regulation of cellular component movement** | **57** |  | **Regulation of cellular component movement** | **54** |
| **Negative regulation of response to stimulus** | **84** |  | **Negative regulation response to stimulus** | **84** |
| **Inflammatory response** | **31** |  | **Inflammatory response** | **31** |
| **Regulation of signal transduction** | **143** |  | **Regulation of signal transduction** | **139** |
|  |  |  | Single-multicellular organismal process | 155 |
|  |  |  | Receptor binding | 67 |
|  |  |  | Negative regulation of signal transduction | 67 |
| Extracellular matrix | 47 |  |  |  |
| Proteinaceous extracellular matrix | 32 |  |  |  |
| Developmental process | 268 |  |  |  |
| G-protein coupled receptor signaling pathway | 40 |  |  |  |
| Signaling receptor binding | 73 |  |  |  |
| Cell surface receptor signaling pathway | 130 |  |  |  |
| Regulation of multicellular organismal development | 104 |  |  |  |
| Negative regulation of cell proliferation | 48 |  |  |  |
| Transmembrane signaling receptor activity | 48 |  |  |  |
| Regulation of developmental process | 136 |  |  |  |
| Anatomical structure morphogenesis | 94 |  |  |  |
| Animal organ development | 84 |  |  |  |
| Signaling receptor activity | 52 |  |  |  |
| Plasma membrane part | 129 |  |  |  |
| Cell surface | 39 |  |  |  |
| Extracellular region part | 183 |  |  |  |
| Animal organ morphogenesis | 34 |  |  |  |
| Regulation of epithelial cell proliferation | 25 |  |  |  |
| Glycosaminoglycan binding | 22 |  |  |  |
| System development | 40 |  |  |  |
| Positive regulation of multicellular organismal process | 81 |  |  |  |
| Embryonic morphogenesis | 34 |  |  |  |
| Organic anion transport | 29 |  |  |  |
| Response to lipid | 50 |  |  |  |
| Cellular developmental process | 149 |  |  |  |
| Response to hormone | 45 |  |  |  |
| Positive regulation of cell proliferation | 48 |  |  |  |
| Negative regulation of multicellular organismal process | 63 |  |  |  |
| Response to endogenous stimulus | 67 |  |  |  |
| Response to oxygen-containing compound | 74 |  |  |  |
| Regulation of vasculature development | 23 |  |  |  |

Again, there is broad conservation of the terms found from the original edgeR analysis with primary differences coming from greater specificity from several terms related to signaling, the response to stimulus, and tissue repair and replacement.

**The shared response between *E. maclovinus* and *C. rastrospinosus***

| **Limma + Voom** | |  | **edgeR** | |
| --- | --- | --- | --- | --- |
| **GO Term** | **DE genes** |  | **GO Term** | **DE Genes** |
| **Regulation of cellular process** | **205** |  | **Regulation of cellular process** | **201** |
| **Regulation of metabolic process** | **147** |  | **Regulation of metabolic process** | **143** |
| **Sequence-specific DNA binding** | **37** |  | **Sequence-specific DNA binding** | **36** |
| **Regulation of macromolecule metabolic process** | **133** |  | **Regulation of macromolecule metabolic process** | **130** |
| **Biological regulation** | **218** |  | **Biological regulation** | **217** |
| **RNA polymerase II transcription factor activity, sequence-specific DNA binding** | **26** |  | **RNA polymerase II transcription factor activity, sequence-specific DNA binding** | **23** |
| **Regulation of cellular metabolic process** | **134** |  | **Regulation of cellular metabolic process** | **131** |
| **Regulation of macromolecule biosynthetic process** | **100** |  | **Regulation of macromolecule biosynthetic process** | **100** |
| **Signal transduction** | **99** |  | **Signal transduction** | **97** |
| **Regulation of biological process** | **208** |  | **Regulation of biological process** | **204** |
| **Intracellular signal transduction** | **53** |  | **Intracellular signal transduction** | **52** |
| **Response to stimulus** | **107** |  | **Response to stimulus** | **102** |
| **Regulation of cellular biosynthetic process** | **100** |  | **Regulation of cellular biosynthetic process** | **101** |
| **Regulation of primary metabolic process** | **127** |  | **Regulation of primary metabolic process** | **127** |
| **Regulation of gene expression** | **104** |  | **Regulation of gene expression** | **105** |
| **Regulation of cellular macromolecule biosynthetic process** | **96** |  | **Regulation of cellular macromolecule metabolic process** | **96** |
| **Regulation of biosynthetic process** | **100** |  | **Regulation of biosynthetic process** | **101** |
| **Regulation of RNA biosynthetic process** | **83** |  | **Regulation of RNA biosynthetic process** | **85** |
|  |  |  | Nucleic acid binding transcription factor activity | 39 |
|  |  |  | Transcription factor activity, sequence-specific dna binding | 39 |
|  |  |  | Reg transcription, dna-templated | 84 |
|  |  |  | Reg nucleic acid-templated transcription | 84 |
| DNA binding transcription factor activity | 42 |  |  |  |
| Negative regulation of biological process | 107 |  |  |  |
| Negative regulation of response to stimulus | 44 |  |  |  |
| Regulation of response to stimulus | 86 |  |  |  |
| Response to external stimulus | 39 |  |  |  |
| Response to lipid | 29 |  |  |  |
| Immune system process | 46 |  |  |  |
| Regulation of apoptotic process | 45 |  |  |  |
| Regulation of programmed cell death | 45 |  |  |  |
| Response to chemical | 62 |  |  |  |
| Response to biotic stimulus | 26 |  |  |  |
| Immune response | 29 |  |  |  |
| Response to external biotic stimulus | 25 |  |  |  |
| Negative regulation of cellular process | 97 |  |  |  |
| Transcription regulatory region sequence-specific DNA binding | 22 |  |  |  |
| Negative regulation of apoptotic process | 29 |  |  |  |
| Regulation of cell proliferation | 42 |  |  |  |
| Negative regulation of signal transduction | 35 |  |  |  |
| Negative regulation of programmed cell death | 29 |  |  |  |

Finally, looking at the shared response we again see that nearly all of the response detected from the edgeR analysis was conserved. Most additional enriched terms again serve to provide greater specificity in areas that already showed enrichment. The enrichment of several terms tied to the regulation of apoptosis was detected in the limma analysis only.

Overall, Limma with Voom normalization shows a differing level of sensitivity but does validate the original response found from the edgeR analysis. The aberrant P-value histogram appears to be an artifact resulting from the lack of biological signal combined perhaps with heteroscedasticity but not an apparent failure to discriminate the biological response as they are conserved between the two distinct approaches of analyses.
