# Supplementary material for: Evolution in chronic cold: varied loss of cellular response to heat in Antarctic notothenioid fish
Source: BMC Evol Biol. 2018 Sep 19;18:143. doi: 10.1186/s12862-018-1254-6 (PMC6146603; doi:10.1186/s12862-018-1254-6)
Supplement: Supplementary file 3 — Investigating the Role of CPM Threshold on the Detected Biological Response. (DOCX 21 kb) [file 12862_2018_1254_MOESM3_ESM.docx]

**Additional file 3,**

**Investigating the Role of CPM Threshold on the Detected Biological Response**

A relatively strict threshold for gene inclusion of CPM>1 in 12 or more libraries was utilized in the primary study. To investigate whether this threshold limited our capacity to identify key differentially expressed (DE) genes we compare the number of DE genes and enriched GO terms using thresholds of 4, 8, and 12 (the study).

first investigating the total number of genes passing these thresholds found only a modest difference as stringency is increased:

**Table 1, Genes Passing the CPM>1 threshold**

| **threshold** | **genes** |
| --- | --- |
| **4 or more libraries** | 13,268 |
| **8 or more libraries** | 12,828 |
| **12 or more libraries** | 12,420 |

This was reflected in the number of differentially expressed genes which were similar across thresholds:

**Table 2, Total Number of Differentially Expressed Genes by Species for Each CPM>1 Threshold**

| **Species** | **4 library threshold** | **8 library threshold** | **12 library threshold** |
| --- | --- | --- | --- |
| ***E. maclovinus*** | 1,527 | 1,500 | 1,481 |
| ***C. rastrospinosus*** | 1,308 | 1,289 | 1,273 |
| ***P. borchgrevinki*** | 29 | 25 | 19 |

Where differentially expressed genes were identified using 1 log_2_FC and FDR adjusted p value 0.01 thresholds as in the study. These again showed only modest differences in differentially expressed genes with the three selected CPM>1 thresholds and confirmed that the stringent threshold was not obscuring a species specific set of responding genes in the case of *P. borchgrevinki*.

GO enrichment was then compared between the three thresholds, looking at the species specific and shared sets of DE genes for *E. maclovinus* and *C. rastrospinosus* which were the focus of investigation in the manuscript. *P. borchgrevinki* failed to show GO enrichment at any of the above thresholds and is thus not shown below.

**Table 3A, Enriched GO Terms in the *E. maclovinus* Specific Response**

| **4 library threshold**  **1,178 genes** | |  | **8 library threshold**  **1,155 genes** | |  | **12 library threshold**  **1,137 genes** | |  |
| --- | --- | --- | --- | --- | --- | --- | --- | --- |
| **GO term** | **#genes** |  | **GO term** | **#genes** |  | **GO term** | **#genes** |  |
| Endoplasmic Reticulum Part | 125 |  | Endoplasmic Reticulum Part | 122 |  | Endoplasmic Reticulum Part | 123 |  |
| Endoplasmic Reticulum Membrane | 97 |  | Endoplasmic Reticulum Membrane | 95 |  | Endoplasmic Reticulum Membrane | 96 |  |
| Protein Folding | 39 |  | Protein Folding | 37 |  | Protein Folding | 37 |  |
| Alcohol Biosynthetic Process | 22 |  | Alcohol Biosynthetic Process | 22 |  | Alcohol Biosynthetic Process | 22 |  |
|  |  |  | Organic Hydroxyl Compound Biosynthetic Process | 22 |  | Organic Hydroxyl Compound Biosynthetic Process | 22 |  |
| Sequence-specific DNA binding | 75 |  |  |  |  |  |  |  |

For the *E. maclovinus* specific response, GO enrichment captures highly similar responses across all three thresholds though capturing an additional term likely related to transcription factor activity in with the four library threshold.

**Table 3B, Enriched GO Terms in the *C. rastrospinosus* Specific Response**

| **4 library threshold**  **960 genes** | |  | **8 library threshold**  **945 genes** | |  | **12 library threshold**  **929 genes** | |  |
| --- | --- | --- | --- | --- | --- | --- | --- | --- |
| **GO term** | **#genes** |  | **GO term** | **#genes** |  | **GO term** | **#genes** |  |
| Single-multicellular organismal process | 161 |  | Single-multicellular organismal process | 158 |  | Single-multicellular organismal process | 155 |  |
| Multicellular organismal process | 162 |  | Multicellular organismal process | 159 |  | Multicellular organismal process | 156 |  |
| Positive reg cell migration | 43 |  | Positive reg cell migration | 40 |  | Positive reg cell migration | 40 |  |
| Positive reg cell motility | 43 |  | Positive reg cell motility | 40 |  | Positive reg cell motility | 40 |  |
| Positive reg cellular component movement | 43 |  | Positive reg cellular component movement | 40 |  | Positive reg cellular component movement | 40 |  |
| Positive reg of locomotion | 43 |  | Positive reg of locomotion | 40 |  | Positive reg of locomotion | 40 |  |
| Reg cell migration | 57 |  | Reg cell migration | 54 |  | Reg cell migration | 54 |  |
|  |  |  | Extracellular region | 62 |  | Extracellular region | 63 |  |
| Neg reg response to stimulus | 85 |  | Neg reg response to stimulus | 84 |  | Neg reg response to stimulus | 84 |  |
| Receptor binding | 69 |  | Receptor binding | 68 |  | Receptor binding | 67 |  |
| Reg of locomotion | 61 |  | Reg of locomotion | 58 |  | Reg of locomotion | 58 |  |
| Reg of cell motility | 58 |  | Reg of cell motility | 54 |  | Reg of cell motility | 54 |  |
| Reg of multicellular organismal process | 138 |  | Reg of multicellular organismal process | 137 |  | Reg of multicellular organismal process | 136 |  |
| Extracellular space | 64 |  | Extracellular space | 61 |  | Extracellular space | 60 |  |
| Reg of signal transduction | 144 |  | Reg of signal transduction | 141 |  | Reg of signal transduction | 139 |  |
| Reg of cell proliferation | 83 |  | Reg of cell proliferation | 82 |  | Reg of cell proliferation | 82 |  |
|  |  |  | G-protein coupled receptor activity | 24 |  | G-protein coupled receptor activity | 24 |  |
| Cell differentiation | 106 |  | Cell differentiation | 103 |  | Cell differentiation | 102 |  |
| Reg cellular component movement | 58 |  | Reg cellular component movement | 54 |  | Reg cellular component movement | 54 |  |
|  |  |  |  |  |  | Inflammatory response | 31 |  |
|  |  |  | Integral component of plasma membrane | 61 |  | Integral component of plasma membrane | 59 |  |
|  |  |  |  |  |  | Neg reg of signal transduction | 67 |  |
|  |  |  | Intrinsic component of plasma membrane | 63 |  | Intrinsic component of plasma membrane | 61 |  |

In contrast to the *E. maclovinus* specific response, the *C. rastrospinosus* specific response captures greater specificity with the most stringent criteria though again the three thresholds produce highly similar sets of enriched GO terms.

**Table 3C, Enriched GO Terms in the shared *E. maclovinus* and *C. rastrospinosus* Response**

| **4 library threshold**  **348 genes** | |  | **8 library threshold**  **344 genes** | |  | **12 library threshold**  **344 genes** | |  |
| --- | --- | --- | --- | --- | --- | --- | --- | --- |
| **GO term** | **#genes** |  | **GO term** | **#genes** |  | **GO term** | **#genes** |  |
| Nucleic acid binding transcription factor activity | 38 |  | Nucleic acid binding transcription factor activity | 39 |  | Nucleic acid binding transcription factor activity | 39 |  |
| Transcription factor activity, sequence-specific DNA binding | 38 |  | Transcription factor activity, sequence-specific DNA binding | 39 |  | Transcription factor activity, sequence-specific DNA binding | 39 |  |
| Biological regulation | 221 |  | Biological regulation | 218 |  | Biological regulation | 217 |  |
| Sequence-specific DNA binding | 35 |  | Sequence-specific DNA binding | 35 |  | Sequence-specific DNA binding | 36 |  |
| Reg cellular process | 204 |  | Reg cellular process | 203 |  | Reg cellular process | 201 |  |
| Reg macromolecule biosynthetic process | 100 |  | Reg macromolecule biosynthetic process | 100 |  | Reg macromolecule biosynthetic process | 100 |  |
| Reg macromolecule metabolic process | 130 |  | Reg macromolecule metabolic process | 130 |  | Reg macromolecule metabolic process | 130 |  |
| Reg cellular biosynthetic process | 101 |  | Reg cellular biosynthetic process | 101 |  | Reg cellular biosynthetic process | 101 |  |
| Reg metabolic process | 143 |  | Reg metabolic process | 143 |  | Reg metabolic process | 143 |  |
| Reg gene expression | 105 |  | Reg gene expression | 105 |  | Reg gene expression | 105 |  |
| Reg biosynthetic process | 101 |  | Reg biosynthetic process | 101 |  | Reg biosynthetic process | 101 |  |
| Signal transduction | 102 |  | Signal transduction | 99 |  | Signal transduction | 97 |  |
| Intracellular signal transduction | 53 |  | Intracellular signal transduction | 52 |  | Intracellular signal transduction | 52 |  |
| Reg primary metabolic process | 127 |  | Reg primary metabolic process | 127 |  | Reg primary metabolic process | 127 |  |
| Reg cellular metabolic process | 131 |  | Reg cellular metabolic process | 131 |  | Reg cellular metabolic process | 131 |  |
| Reg cellular macromolecule metabolic process | 96 |  | Reg cellular macromolecule metabolic process | 96 |  | Reg cellular macromolecule metabolic process | 96 |  |
| RNA polymerase II transcription factor activity, sequence-specific DNA binding | 22 |  | RNA polymerase II transcription factor activity, sequence-specific DNA binding | 23 |  | RNA polymerase II transcription factor activity, sequence-specific DNA binding | 23 |  |
| Reg RNA biosynthetic process | 85 |  | Reg RNA biosynthetic process | 85 |  | Reg RNA biosynthetic process | 85 |  |
| Reg biological process | 207 |  | Reg biological process | 206 |  | Reg biological process | 204 |  |
| Reg transcription, DNA-templated | 84 |  | Reg transcription, DNA-templated | 84 |  | Reg transcription, DNA-templated | 84 |  |
| Reg nucleic acid-templated transcription | 84 |  | Reg nucleic acid-templated transcription | 84 |  | Reg nucleic acid-templated transcription | 84 |  |
| Response to stimulus | 106 |  | Response to stimulus | 104 |  | Response to stimulus | 102 |  |
| Integral component of plasma membrane | 34 |  | Integral component of plasma membrane | 33 |  |  |  |  |
| Reg RNA metabolic process | 85 |  | Reg RNA metabolic process | 87 |  |  |  |  |
| Reg nitrogen compound metabolic process | 97 |  | Reg nitrogen compound metabolic process | 97 |  |  |  |  |
| DNA binding | 59 |  | DNA binding | 59 |  |  |  |  |

Finally, the shared set does show slightly greater specificity with reduced stringency though nearly all enriched terms are shared across the three thresholds.

Ultimately these show that the chosen CPM library threshold did not limit our view of the biological response in the study species or prevent us from identifying a response in *P. borchgrevinki*.
