# Supplementary material for: Evolution in chronic cold: varied loss of cellular response to heat in Antarctic notothenioid fish
Source: BMC Evol Biol. 2018 Sep 19;18:143. doi: 10.1186/s12862-018-1254-6 (PMC6146603; doi:10.1186/s12862-018-1254-6)
Supplement: Supplementary file 4 — Investigating the Role of the FDR Threshold on the Detected Biological Response. (DOCX 19 kb) [file 12862_2018_1254_MOESM4_ESM.docx]

**Additional file 4,**

**Investigating the Role of the FDR Threshold on the Detected Biological Response**

We applied a 1 log_2_FC and 0.01 FDR adjusted p-value threshold for determining differentially expressed genes. To explore whether the strict FDR adjusted p-value threshold has limited our ability fully observe the biological response, especially in *P. borchgrevinki* where the original response was minimal, we repeated the analysis with a 0.05 threshold.

**Table 1, Differentially Expressed Genes**

| **Species** | **0.05 threshold** | **0.01 threshold** |
| --- | --- | --- |
| ***E. maclovinus*** | 1,607 | 1,481 |
| ***C. rastrospinosus*** | 1,410 | 1,273 |
| ***P. borchgrevinki*** | 25 | 19 |

This produces a relatively modest change in differentially expressed genes and again fails to identify a meaningful response in *P. borchgrevinki*. To determine though whether this results in a change in the measured biological response we again carried out GO enrichment analysis focusing on the *E. maclovinus* specific, *C. rastrospinosus* specific, and their shared responses as in the main text.

**Table 2A, Enriched GO Terms in the *E. maclovinus* Specific Response**

| **0.05 threshold**  **1,217 genes** | |  | **0.01 threshold**  **1,139 genes** | |  |
| --- | --- | --- | --- | --- | --- |
| **GO term** | **# DE genes** |  | **GO term** | **# DE genes** |  |
| endoplasmic reticulum part | 125 |  | endoplasmic reticulum part | 123 |  |
| endoplasmic reticulum membrane | 97 |  | endoplasmic reticulum membrane | 96 |  |
| protein folding | 36 |  | protein folding | 37 |  |
| alcohol biosynthetic process | 23 |  | alcohol biosynthetic process | 22 |  |
| small molecule biosynthetic process | 46 |  |  |  |  |
| organic hydroxy compound biosynthetic process | 23 |  | organic hydroxy compound biosynthetic process | 22 |  |

**Table 2B, Enriched GO Terms in the *C. rastrospinosus* Specific Response**

| **0.05 threshold**  **1,020 genes** | |  | **0.01 threshold**  **929 genes** | |  |
| --- | --- | --- | --- | --- | --- |
| **GO term** | **# DE genes** |  | **GO term** | **# DE genes** |  |
| single-multicellular organism process | 165 |  | single-multicellular organism process | 155 |  |
| multicellular organismal process | 166 |  | multicellular organismal process | 156 |  |
| positive regulation of cell migration | 41 |  | positive regulation of cell migration | 40 |  |
| positive regulation of cell motility | 41 |  | positive regulation of cell motility | 40 |  |
| positive regulation of cellular component movement | 41 |  | positive regulation of cellular component movement | 40 |  |
| positive regulation of locomotion | 41 |  | positive regulation of locomotion | 40 |  |
| regulation of cell migration | 55 |  | regulation of cell migration | 54 |  |
| extracellular region | 69 |  | extracellular region | 63 |  |
| negative regulation of response to stimulus | 88 |  | negative regulation of response to stimulus | 84 |  |
| receptor binding | 71 |  | receptor binding | 67 |  |
| regulation of locomotion | 59 |  | regulation of locomotion | 58 |  |
| regulation of cell motility | 55 |  | regulation of cell motility | 54 |  |
| regulation of multicellular organismal process | 148 |  | regulation of multicellular organismal process | 136 |  |
| extracellular space | 63 |  | extracellular space | 60 |  |
|  |  |  | regulation of signal transduction | 139 |  |
| regulation of cell proliferation | 88 |  | regulation of cell proliferation | 82 |  |
| G-protein coupled receptor activity | 26 |  | G-protein coupled receptor activity | 24 |  |
|  |  |  | cell differentiation | 102 |  |
|  |  |  | regulation of cellular component movement | 54 |  |
|  |  |  | inflammatory response | 31 |  |
| integral component of plasma membrane | 63 |  | integral component of plasma membrane | 59 |  |
|  |  |  | negative regulation of signal transduction | 67 |  |
|  |  |  | intrinsic component of plasma membrane | 61 |  |
| proteinaceous extracellular matrix | 27 |  |  |  |  |
| Extracellular matrix | 39 |  |  |  |  |
| intrinsic component of plasma membrane | 65 |  |  |  |  |
| Cell surface | 39 |  |  |  |  |

**Table 2C, Enriched GO Terms in the Shared Response Between *E. maclovinus* and *C. rastrospinosus***

| **0.05 threshold**  **390** | |  | **0.01 threshold**  **344 genes** | |  |
| --- | --- | --- | --- | --- | --- |
| **GO term** | **# DE genes** |  | **GO term** | **# DE genes** |  |
| nucleic acid binding transcription factor activity | 42 |  | nucleic acid binding transcription factor activity | 39 |  |
| transcription factor activity, sequence-specific DNA binding | 42 |  | transcription factor activity, sequence-specific DNA binding | 39 |  |
| biological regulation | 246 |  | biological regulation | 217 |  |
| sequence-specific DNA binding | 38 |  | sequence-specific DNA binding | 36 |  |
| regulation of cellular process | 226 |  | regulation of cellular process | 201 |  |
| regulation of macromolecule biosynthetic process | 107 |  | regulation of macromolecule biosynthetic process | 100 |  |
| regulation of macromolecule metabolic process | 143 |  | regulation of macromolecule metabolic process | 130 |  |
| regulation of cellular biosynthetic process | 145 |  | regulation of cellular biosynthetic process | 101 |  |
| regulation of metabolic process | 160 |  | regulation of metabolic process | 143 |  |
| regulation of gene expression | 113 |  | regulation of gene expression | 105 |  |
|  |  |  | regulation of biosynthetic process | 101 |  |
| signal transduction | 106 |  | signal transduction | 97 |  |
| intracellular signal transduction | 55 |  | intracellular signal transduction | 52 |  |
|  |  |  | regulation of primary metabolic process | 127 |  |
|  |  |  | regulation of cellular metabolic process | 131 |  |
| regulation of cellular macromolecule biosynthetic process | 103 |  | regulation of cellular macromolecule biosynthetic process | 96 |  |
| RNA polymerase II transcription factor activity, sequence-specific DNA binding | 25 |  | RNA polymerase II transcription factor activity, sequence-specific DNA binding | 23 |  |
|  |  |  | regulation of RNA biosynthetic process | 85 |  |
| regulation of biological process | 230 |  | regulation of biological process | 204 |  |
|  |  |  | regulation of transcription, DNA-templated | 84 |  |
|  |  |  | regulation of nucleic acid-templated transcription | 84 |  |
| response to stimulus | 119 |  | response to stimulus | 102 |  |
| regulation of cellular biosynthetic process | 109 |  |  |  |  |
| regulation of biosynthetic process | 109 |  |  |  |  |
| response to lipid | 32 |  |  |  |  |
| regulation of primary metabolic process | 137 |  |  |  |  |
| negative regulation of biological process | 114 |  |  |  |  |
| developmental process | 125 |  |  |  |  |

Though these do differ between thresholds, when the analysis was run using an 0.05 FDR adjusted p value cutoff the downstream suite of enriched GO terms was overwhelmingly similar to what was seen when using the 0.01 cutoff. These differed primarily in general or redundant GO terms suggesting minimal information gain with the small increase in differentially expressed genes. Further, this shows that the P-value threshold was not itself responsible for obscuring a response in *P. borchgrevinki*.
