# Supplementary figures and images for: Evolution in chronic cold: varied loss of cellular response to heat in Antarctic notothenioid fish
Source: BMC Evol Biol. 2018 Sep 19;18:143. doi: 10.1186/s12862-018-1254-6 (PMC6146603; doi:10.1186/s12862-018-1254-6)

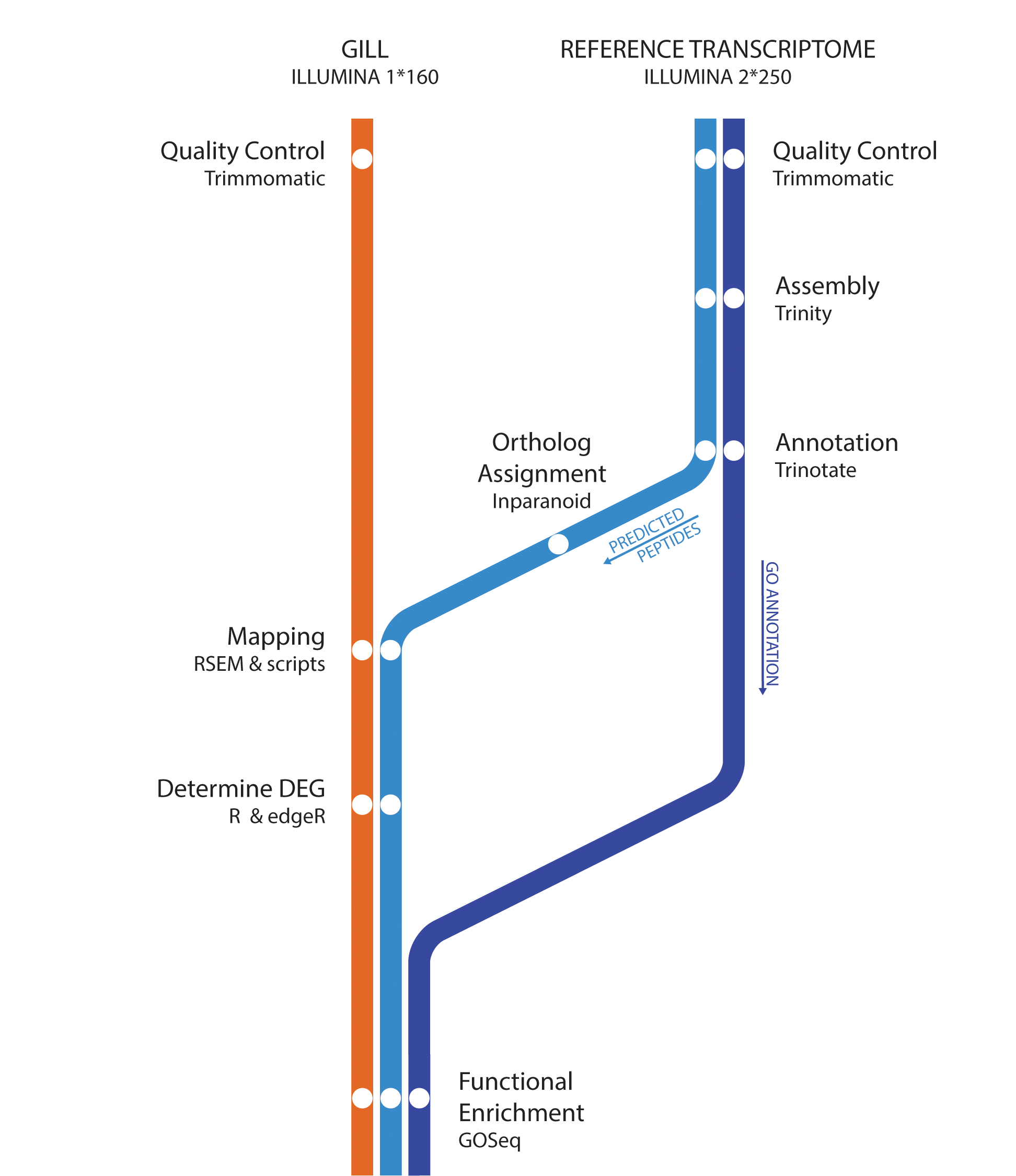

Supplement: Supplementary file 5 — Figure S1. Overview of the bioinformatics workflow used in the analysis. (TIF 13925 kb) [file 12862_2018_1254_MOESM5_ESM.tif]

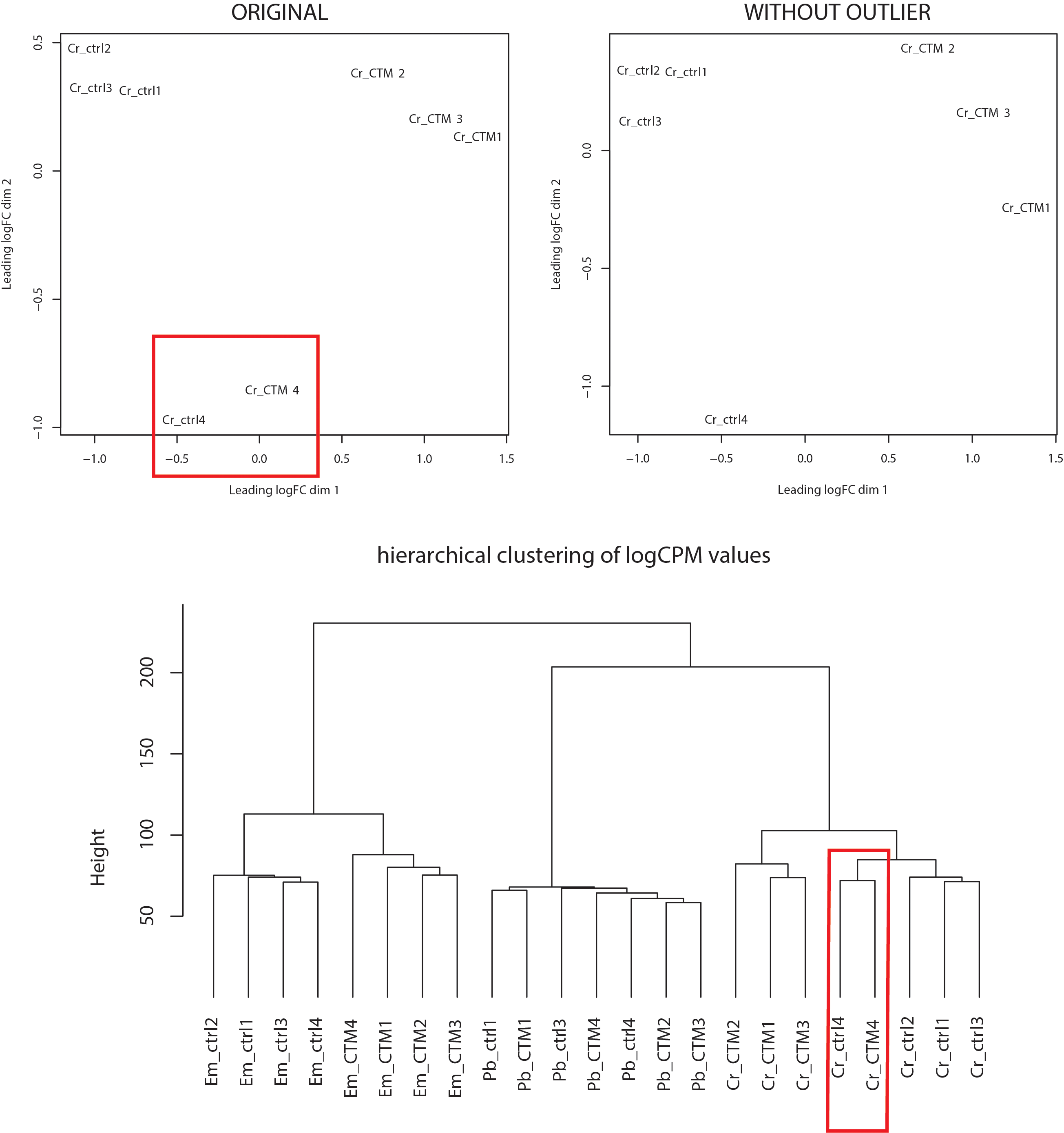

Supplement: Supplementary file 7 — Figure S2. MDS plot and Hierarchical Clustering analysis used to identify the C. rastrospinosus outlier. (TIF 22467 kb) [file 12862_2018_1254_MOESM7_ESM.tif]
