# Supplementary material for: Evolution in chronic cold: varied loss of cellular response to heat in Antarctic notothenioid fish
Source: BMC Evol Biol. 2018 Sep 19;18:143. doi: 10.1186/s12862-018-1254-6 (PMC6146603; doi:10.1186/s12862-018-1254-6)
Supplement: Supplementary file 6 — Trimmomatic Settings. (DOCX 19 kb) [file 12862_2018_1254_MOESM6_ESM.docx]

**Additional file 6, Trimmomatic Settings**

**Supplementary Material 1,**

**Trimmomatic Settings**

**For the reference transcriptomes:**

ILLUMINACLIP:<filename>:2:30:10 LEADING:20 TRAILING:20 SLIDINGWINDOW:4:15 MINLEN:70

**For the individual Gill libraries:**

ILLUMINACLIP <filename> 4:25:6 LEADING 20 TRAILING 20 SLIDINGWINDOW 3:20 HEADCROP 5 CROP 140 MINLEN 70
