# Supplementary material for: Evolution in chronic cold: varied loss of cellular response to heat in Antarctic notothenioid fish
Source: BMC Evol Biol. 2018 Sep 19;18:143. doi: 10.1186/s12862-018-1254-6 (PMC6146603; doi:10.1186/s12862-018-1254-6)
Supplement: Supplementary file 8 — Evaluating the Biological Content of the Residual Reads. (DOCX 29 kb) [file 12862_2018_1254_MOESM8_ESM.docx]

**Additional file 8,**

**Evaluating the Content of the Residual Reads**

While the orthologous sets did account for the majority of sequenced reads, they did not account for all of the reads that could map to the original species specific transcriptomes. These unmapped reads could contain signs of a biological response that was not captured in the orthologous response, thus making them of interest. To test for potential unaccounted for response, we mapped the residual reads against the leftover segment of its species transcriptome, that is, the portion of the transcriptome that was not accounted for in the orthologous set.

The residual reads were originally isolated using bowtie2 as the unmapped portion of each library when mapped against the orthologous set. To remove redundancy, each species leftover set was first condensed using CD-Hit-Est with a 99% threshold. As with the original read counting, these were mapped to its species’ leftover set, again using bowtie2, and gene counts estimated using RSEM. Trinity gene counts were then compared between heat stressed and control specimens by species using edgeR with 0.01 FDR and one logFC thresholds.

|  | *E. maclovinus* | *C. rastrospinosus* | *P. borchgrevinki* |
| --- | --- | --- | --- |
|  |  |  |  |
| Genes | 108,688 | 121,187 | 103,963 |
| Tested Genes | 25,818 | 42,784 | 34,039 |
| Differentially Expressed | 1,543 | 481 | 79 |

From these we again see the same broad pattern detected using the orthologous set. As before, the largest number of differentially expressed (DE) genes were found in the *E. maclovinus* response, *C. rastrospinosus* had a smaller, though still substantial response, while *P. borchgrevinki* had an incredibly muted transcriptional response. To place these differentially expressed genes into a functional context we then analyzed them for Enriched Gene Ontology groups using GOSeq with an FDR threshold of 0.05 and excluding any terms with fewer than 20 underlying DE genes. The resulting enrichment analysis followed the trends in differential expression and are described below.

The largest set of enriched terms in *E. maclovinus* (20):

| **Category** | **Description** | **# DE genes** | **FDR** |
| --- | --- | --- | --- |
|  |  |  |  |
| GO:0006457 | protein folding | 51 | 1.51e-26 |
| GO:0006950 | response to stress | 148 | 7.73e-06 |
| GO:0044432 | endoplasmic reticulum part | 73 | 2.63e-04 |
| GO:0050896 | response to stimulus | 206 | 2.17e-03 |
| GO:0042221 | response to chemical | 121 | 2.31e-03 |
| GO:0002376 | immune system process | 95 | 2.67e-03 |
| GO:0030554 | adenyl nucleotide binding | 125 | 4.42e-03 |
| GO:0032559 | adenyl ribonucleotide binding | 124 | 5.68e-03 |
| GO:0005524 | ATP binding | 122 | 5.68e-03 |
| GO:0005783 | endoplasmic reticulum | 60 | 5.68e-03 |
| GO:0044267 | cellular protein metabolic process | 165 | 6.93e-03 |
| GO:0051249 | regulation of lymphocyte activation | 28 | 1.50e-02 |
| GO:0019538 | protein metabolic process | 194 | 1.64e-02 |
| GO:0002683 | negative regulation of immune system process | 27 | 2.03e-02 |
| GO:0044238 | primary metabolic process | 364 | 2.87e-02 |
| GO:0005789 | endoplasmic reticulum membrane | 52 | 2.87-02 |
| GO:0002696 | positive regulation of leukocyte activation | 22 | 3.55e-02 |
| GO:0006952 | defense response | 54 | 3.79e-02 |
| GO:0043167 | ion binding | 326 | 3.85e-02 |
| GO:0050867 | positive regulation of cell activation | 23 | 3.91e-02 |

The enriched GO terms suggest somewhat better resolution of the response to stress and the response to heat, as well as a novel impact on immune response, but are generally in broad agreement with the response seen with read mapping to the orthologous gene set, showing an expected functional response to acute heat shock.

By comparison, while *C. rastrospinosus* continued to demonstrate some enrichment, this was substantially below that seen in *E. maclovinus*:

| **Category** | **Description** | **# DE genes** | **FDR** |
| --- | --- | --- | --- |
|  |  |  |  |
| GO:0005615 | extracellular space | 21 | 9.13e-04 |
| GO:0016788 | hydrolase activity, acting on ester bonds | 23 | 3.04e-03 |

Of the two enriched terms, extracellular space was in keeping with one of the major functional trends discerned from read mapping to the orthologous set. The generally reduced number of differentially expressed genes found from the icefish residual reads may suggest that much of the transcriptional signal of its response was in fact captured in the orthologous set.

Finally, the small number of differentially expressed genes in *P. borchgrevinki* did not show any significant functional enrichment.

Altogether, the analysis of the residual reads does help to demonstrate that while the orthologous set could not account for all the sequenced reads, the remainder of the transcriptional signal do not present a substantially differing transcriptional response.
